# Nonadult Supervision of Children in Low- and Middle-Income Countries: Results from 61 National Population-Based Surveys

**DOI:** 10.3390/ijerph15081564

**Published:** 2018-07-24

**Authors:** Mónica Ruiz-Casares, José Ignacio Nazif-Muñoz, René Iwo, Youssef Oulhote

**Affiliations:** 1 Department of Psychiatry, McGill University, and SHERPA-Institut Universitaire, Centre Intégré Universitaire de Santé et de Services Sociaux du Centre-Ouest-de-l’Île-de-Montréal, Montreal, QC H1T 2M4, Canada; 2 Institute for Health and Social Policy, McGill University, Montreal, QC H3A 1A3, Canada; jose.nazifmunoz@mail.mcgill.ca (J.I.N.-M.); rene.iwo@mail.mcgill.ca (R.I.); 3 Harvard T. H. Chan School of Public Health, Boston, MA 02115, USA; youlhote@hsph.harvard.edu

**Keywords:** child supervision, home alone, low- and middle-income countries, Multiple Indicator Cluster Survey (MICS), Demographic and Health Surveys (DHS)

## Abstract

Despite scarce empirical research in most countries, evidence has shown that young children are unsupervised or under the supervision of another young child while their adult caregivers attend work or engage in other activities outside the home. Lack of quality supervision has been linked to unintentional childhood injuries and other negative outcomes. Nationally representative, population-based data from rounds four and five of the Multiple Indicator Cluster Surveys (MICS) and four to eight of the Demographic and Health Surveys (DHS) from 61 low- and middle-income countries were used to estimate prevalence and socio-economic factors associated with leaving children under five years old home alone or under the care of another child younger than 10 years of age. Socio-economic factors included age and sex of the child, rurality, wealth, maternal education, and household composition. Large variations in the prevalence rates (0.1–35.3% for children home alone and 0.2–50.6% for children supervised by another child) and associated factors have been recorded within and across regions and countries. Understanding why and under what conditions children are home alone or under the supervision of another child is crucial to the development of suitable policies and interventions to protect young children, promote healthy growth, and support caregivers.

## 1. Introduction

Adequate supervision sets the basis for healthy physical and emotional development and is important for the prevention of injuries, particularly among young children [1,2,3]. Unintentional injuries are a leading cause of childhood death and disability worldwide, particularly after the age of one year [4]. Children in low- and middle-income countries and poorer children in high-income countries are the most vulnerable, with the causes of injury differing by age group [4,5]. Despite variations in the definitions and measures of supervision, lower levels of adult supervision have been associated with higher risk of injuries and of more severe injuries in young children [6,7,8]. Lapses or the absence of supervision have also been linked to antisocial and risky behaviors [9,10,11], poorer school performance [12,13,14,15], sexual abuse [16], and other harmful consequences for children [14].

Around the world, children routinely spend time unsupervised by adults—at times with other children, at times alone. Sibling caretaking is a common practice in many societies that normally occurs within earshot of adults [17,18,19,20]. By participating in caretaking and other household chores, children allow parents to participate in employment and other activities [21,22], are socialized into culturally normative roles [20,23], and strengthen sibling ties [19,22,24,25]. However, negative consequences for child development and schooling for both caretaking [26] and looked after [27] children have also been documented. For instance, researchers have found that children who are supervised by older siblings have a higher risk of injuries than children who are supervised by their parents [28,29,30]. Sometimes, children are too young to perform child care safely. More than half of the families interviewed by Ruiz-Casares and Heymann [31] in Botswana, Mexico, and Vietnam relied on children to help care for other children, and a similar proportion was harmed or faced emergencies while their parents were away. Some caretakers were as young as three years of age. Similarly, Fouts et al.’s [21] study among Bofi farmers and foragers in the Central African Republic documented how farmer children are put under the care of older siblings after weaning and are expected to care for younger siblings by the age of four to five years. There is also evidence that many children stay home alone on a regular or occasional basis [22,31,32]. These “latchkey” children are those who carry the house key as they regularly spend time unsupervised by adults before or after school. Population-based studies with school-age children in the U.S. have found a child’s age to be highly correlated with being home alone. Parental employment also influences being home alone. Other factors, such as the child’s sex, maternal education, household income, and the presence of other adults and children in the household, [32,33] do not consistently increase the likelihood of children being home alone [32,34]. Thus, some studies have found that boys are slightly more likely than girls to be home alone, yet this association was not significant for all age groups [32,35]. Similarly, some evidence has shown that higher maternal education is associated with a higher likelihood of children being home alone, particularly when the mother has at least a college degree [32,35], yet this association does not hold for children over nine years of age [36]. Whereas Vandivere et al. [32] found home alone to be more common in high-income families (even if both low- and high-income groups spent about the same amount of time home alone), other researchers have found no significant association after controlling for other factors (e.g., parents’ perceived neighborhood safety [34]). Finally, some studies have found that children are more likely to be home alone in single parent families [32,37] and the presence of additional adults in the household who are not the parent’s spouse or partner does not decrease the likelihood of home alone [32]. Other researchers, however, did not find a relationship between the number of adults in a home and the likelihood [33] or the duration [38] of home alone. Some of these conclusions may, however, result from lack of consideration of confounding factors such as family socio-economic status and neighborhood safety. Regarding children, home alone is more likely to happen when there are no other children under 13 years in the household or when there is at least one child aged 13–17 years; having teenagers in the household, however, does not increase the likelihood of self-care for 10–12-year-olds [32].

The literature on children home alone, however, is rather outdated, with very few studies published in the last decade and from low- and middle-income countries (LMIC). This lack of research interferes with appropriate responses and a clear understanding of child supervision practices. Population-based data on child supervision is very scarce in LMIC and existing studies on children home alone do not include children as young as newborns. To respond to this gap, in the mid 2000’s, UNICEF added optional items on child supervision to the Early Childhood Development Module of the Multiple Indicator Cluster Surveys (MICS). The items inquire about whether a child younger than 5 years has been home alone or under the supervision of another child under 10 years of age for more than an hour in the previous week and, if yes, for how many days of the week. The latest rounds of Demographic and Health Surveys (DHS) also included identical items in some countries. These data have, however, rarely been systematically analyzed, thus further limiting the use of existing evidence. This paper presents data on nonadult supervision of children from 61 standardized national surveys conducted between 2010 and 2016. First, we assess the prevalence of leaving young children home alone or with another child under the age of 10 years in selected LMIC. (Two countries in our sample (i.e., Barbados and Uruguay) are actually classified as High Income Countries (HIC).) Second, we examine whether each of the two types of supervision practices was related to selected child, caregiver, household, and socio-economic status factors within each country.

## 2. Materials and Methods

### 2.1. Data Sources

Data from 52 MICS and nine DHS were used to study supervision practices for children under the age of five. Both standardized, nationally representative, population-based household surveys were implemented in LMIC and provide a unique source of information to monitor the status of children’s wellbeing in different regions of the world. In place since 1995, the MICS were originally administered every five years, and every three years since 2007. To date, more than 100 countries have participated in the MICS program over six rounds, approximately 60 countries per round [39]. Since 1984, more than 300 DHS have been carried out in more than 90 countries over seven phases [40]. MICS and DHS employ a multistage probability sampling and adhere to the fundamentals of scientific sampling, including complete coverage of the target population, the need to conduct a new household listing and pre-selection of sample households, and preparation of appropriate sample documentation [41].

We included all surveys that collected data between 2010 and 2016, were administered to a nationally representative sample, had publicly available datasets in January 2018, and contained the optional items on child supervision according to the version introduced in MICS 4 (see measures of nonadult supervision section). Previous waves of MICS and DHS in several countries either did not have the items of interest or had different wording making the comparison across countries unfeasible. In each country, the last wave of data collection was selected to have the most updated information. Datasets and questionnaires were obtained from UNICEF (http://mics.unicef.org/) and MEASURE DHS (https://dhsprogram.com/). Table 1 shows survey years and sample sizes.

### 2.2. Measures of Nonadult Supervision

Two measures of interest were derived from a question that asks mothers or caregivers the extent to which a randomly selected child aged under five years was at home alone (hereafter referred to as “child home alone”) and supervised by another child younger than 10 years old (“child supervised by another child”). Specifically, the question asked was: “Sometimes adults taking care of children have to leave the house to go shopping, wash clothes, or for other reasons and have to leave young children. On how many days in the past week was (name): (A) Left alone for more than an hour? (B) Left in the care of another child, that is, someone less than 10 years old, for more than an hour?”

Interviewers were trained not to convey judgement in the formulation of the question in order to avoid biased responses. Values of the two measures of child supervision ranged between 0 and 7 in most countries, which represented the number of days in which the child was in each type of nonadult supervision in the week preceding the survey.

### 2.3. Predictors of Nonadult Supervision

The analyses included measures of several household, household member, and child characteristics (Table S1 in Supplementary Materials). Information about children under age five was provided by their mothers or caregivers in the case of MICS, and by biological mothers for DHS. Residence, mother’s educational level, socioeconomic status, and number of individuals in the household were the main variables of interest. Sex and age (in years) of the child were also considered. Residence was a dichotomous variable defined by the survey teams in each country as urban or rural. Mother’s educational level was a dichotomous variable that captured the first two levels of education, which depending on the country could be none and/or primary, and then a second level which could be secondary or higher (Table S2). Mother’s education was thus classified as “low education” and “high education”. This operationalization aimed to facilitate comparison between countries since countries present heterogeneity in terms of both how education is formally organized and the distribution of mothers’ education in each level of education. For instance, a country may have three different types of post-secondary education, whereas another country may have two or one, and these levels had to be grouped to allow cross-country comparison. This operationalization also overcomes conditions in which low values in one category may present problems in statistical operations. For instance, in some countries the number of mothers who are not educated was so low that the distribution of the outcomes of interest was not adequately represented when the associations between these variables were analyzed. 

We assessed socioeconomic status using the Wealth Index Score (WIS) measured in each survey. The WIS incorporates household characteristics (i.e., electricity, water facilities, number of rooms, type of toilet, and building material of roofs, floors, walls), presence of material goods (i.e., television, telephone, refrigerator) and ownership of a computer, camera, and bank account, among other goods. This index is divided into five equal quintiles in which the lowest quintile represents the poorest group and the highest quintile represents the richest. The number of individuals in the household was divided into two variables for the outcome child alone and three variables for the outcome child supervised by another child. The first was the total number of individuals aged 15 years and older who live in the household, which ranged from 1 to 35 depending on the country. Second was the total number of children aged 10 to 14 years living in the household, with a range of 0 to 14. Finally, to determine whether sex was associated with a child supervised by another child, and only for that outcome, we introduced the variable number of girls aged 10 to 14 years, with values ranging from 0 to 8. Due to problems of convergence, the following countries were excluded from some analyses. (1) When predicting child home alone: Argentina and Panama presented problems with all the variables; Barbados, with the variables sex, number of children, wealth, and mother’s education; Belize and Cuba, with the variable wealth; and Serbia, with the variables number of children, mother’s education, and wealth. (2) When predicting child supervised by another child: Argentina presented problems with all the variables; Barbados, with the variables sex, number of children, number of girls, number of adults, mother’s education, wealth, and rurality; Cuba, with the variable wealth; and Saint Lucia, with the variables number of children, number of girls, mother’s education, wealth, and rurality.

### 2.4. Statistical Analyses

Due to the hierarchical structure of the data and the operationalization of our two dependent variables, we employed a two-level random-effect Poisson regression analysis [42,43], with households in the first level and neighborhoods in the second one. This approach allowed us to account for variation due to these levels and to correctly estimate the variance in the parameters of interest [44].

To investigate associations between predictors and outcomes, we derived a separate set of sufficient confounders for each predictor using a priori knowledge and directed acyclic graphs (DAGs) [45]. We chose this strategy to avoid the “Table 2 fallacy” [46] that occurs when investigating multiple predictors using a single multivariable model. Such models do not provide unbiased total effect estimates for the predictors of interest, since some of the covariates may be on the causal pathway between the predictor of interest and outcome. Tables S2, S3, S5, and S6 provide the statistics per country of each covariate included in the models. All analyses used sampling weights to adjust for variation in the probability of selection and were performed using Stata 14 [47].

### 2.5. Ethical Approval

Ethics approval was obtained from the McGill University Faculty of Medicine Institutional Review Board (A09-E84-09B).

## 3. Results

### 3.1. Prevalence of Child Home Alone

**Figure 1:**
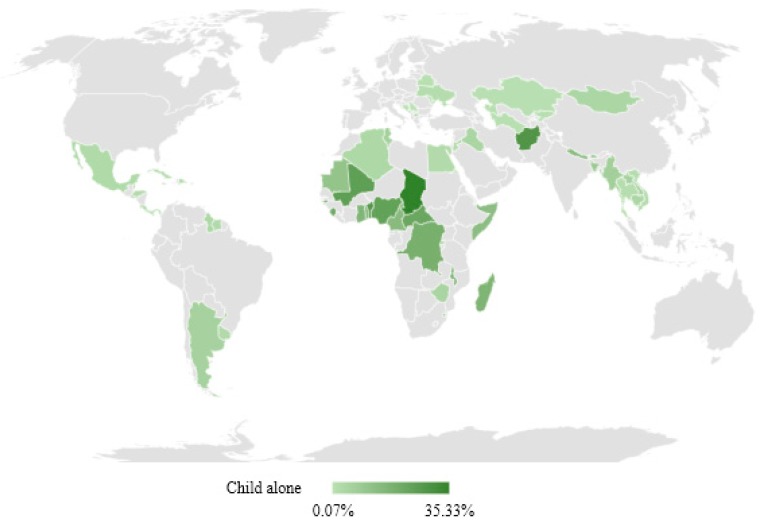
Prevalence of child under five years home alone.

Figure 1 and Table 1 show the reported prevalence of children home alone for at least one hour during the week prior to the survey across 61 countries. In the East Asia and Pacific region (EAP), we observed that between 1.5% (Thailand) and 6.7% (Myanmar) children were home alone. Importantly, Myanmar also had a high prevalence in the number of days during which children were home alone. More than 3.2% children had been home alone for more than three days per week.

In Eastern and Southern Africa (ESA), we observed a higher prevalence of child home alone, ranging between 4.8% in Zimbabwe and 17.4% in Malawi. In this region, high percentages of child home alone more than three days in a week were also observed. Madagascar and Malawi had the highest reported prevalence with 11.0% and 7.8%, respectively.

In Europe and Central Asia (ECA), we found results similar to EAP. The lowest prevalence was in Serbia (0.1%) and the highest was in Kosovo (4.1%). Countries in this region had a very low prevalence of child home alone for more than three days per week. Except for Kosovo (1.1%), all countries displayed values lower than 1%.

In Latin America and the Caribbean (LAC), results were similar to EAP. The lowest prevalence was 0.8% (Belize) and the highest was 5.7% (Argentina). All English-speaking countries in the region, except Guyana (3.6%) and Saint Lucia (2.7%), had a prevalence around 1% or lower. Similar to ECA, most LAC countries had a very low prevalence of child home alone for more than three days in a week. Exceptions to this finding were Argentina, Guyana, and Uruguay.

Countries from the Middle East and North Africa region (MENA) also displayed a low prevalence, ranging from 2.2% (Jordan) to 6.1% (Tunisia). The range in prevalence corresponding to child home alone more than three days per week was also very low.

In South Asia (SA), we observed a high prevalence of child home alone more than once per week, ranging from 6.7% (Bhutan) to 28.5% (Afghanistan). Values associated with child home alone more than three days per week were also relatively higher than ECA and LAC, ranging from 2.7% in Bhutan to 11.1% in Afghanistan.

Finally, the West and Central Africa region (WCA) displayed the highest prevalence of child home alone. The lowest prevalence was observed in Sao Tome and Principe (7.5%) and the highest—the highest in our sample—in Chad (35.3%). In terms of children being home alone for more than three days, this region also had the highest values of the 61 countries analyzed. More specifically, Guinea-Bissau and Chad had a prevalence of 16.7% and 20.3% in this category, respectively.

### 3.2. Prevalence of Child Supervised by Another Child

**Figure 2:**
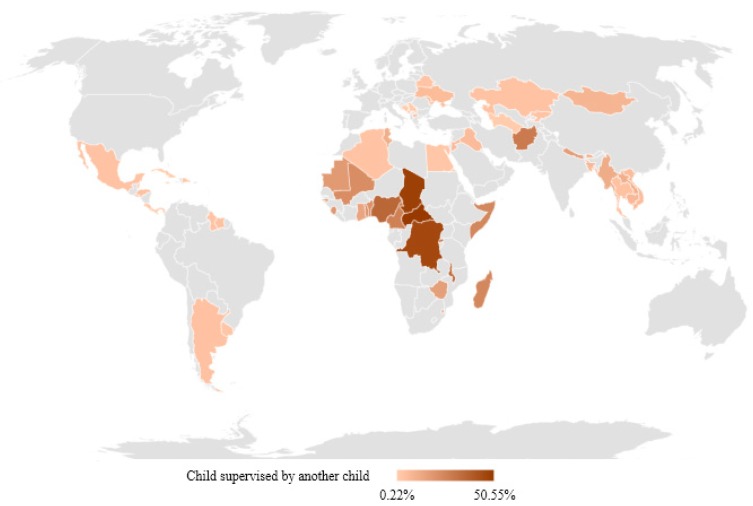
Prevalence of child under five years supervised by another child aged 10 years or younger.

Figure 2 and Table 1 show the reported prevalence of child supervised by another child across 61 countries. These values were generally higher than those of child home alone in the same countries. The exceptions to this trend were Kosovo and Turkmenistan from ECA; Argentina, Barbados, Cuba, El Salvador, Guyana, Jamaica, and Uruguay in LAC; Algeria and Egypt in MENA; Bangladesh in SA; and Benin, Ghana, Guinea-Bissau, Mali, and Sierra Leone in WCA.

In EAP, we observed that between 3.8% (Thailand) and 11.4% (Myanmar) of children had been supervised by another child. Like child home alone results, Myanmar had the highest prevalence and Thailand the lowest. Myanmar and Laos also had a high prevalence (5.9% and 5.4%, respectively) of children being supervised by another child more than three days per week.

In ESA, the range and frequencies for this variable were much larger than EAP, ranging from 10.8% (Swaziland) to 33.8% (Malawi). In this region, a high prevalence of a child supervised by another child more than three days in a week was also observed. Rwanda and Malawi reported the highest prevalence with 20.3% and 17.4%, respectively.

In ECA, the lowest value recorded was 0.5% in Turkmenistan and the highest was 6.0% in Ukraine. Countries in this region had a very low prevalence in terms of leaving a child supervised by another child more than three days per week. Except for Ukraine, all countries displayed values under 2%.

In LAC, results were similar to EAP. The lowest prevalence was 0.2% (Barbados) and the highest was 4.3% (Honduras). Most English-speaking LAC countries had a prevalence lower than 2%. Similar to ECA, most countries in this region had a very low prevalence of a child supervised by another child more than three days per week.

Countries in MENA also displayed a relatively low prevalence, ranging from 2.0% (Egypt) to 11.5% (Palestine). The range of prevalence of a child supervised by another child more than three days per week was also very low.

In SA, we observed a high prevalence of child supervised by another child more than one day per week, ranging from 8.0% (Bangladesh) to 29.0% (Afghanistan). The prevalence of a child supervised by another child more than three days per week was also high, ranging from 4.1% (Bhutan) to 14.9% (Afghanistan).

Lastly, WCA displayed the highest prevalence of a child supervised by another child. The lowest prevalence was observed in Sao Tome and Principe (13.6%) and the highest, which was the highest of our sample, was in Central African Republic (50.6%). In terms of a child supervised by another child more than three days per week, this region had the highest prevalence of the 61 countries analyzed. More specifically, Congo Democratic Republic and Central African Republic had a prevalence of 33.5% and 37.0% in this category, respectively.

### 3.3. Child Age and Sex

**Figure 3:**
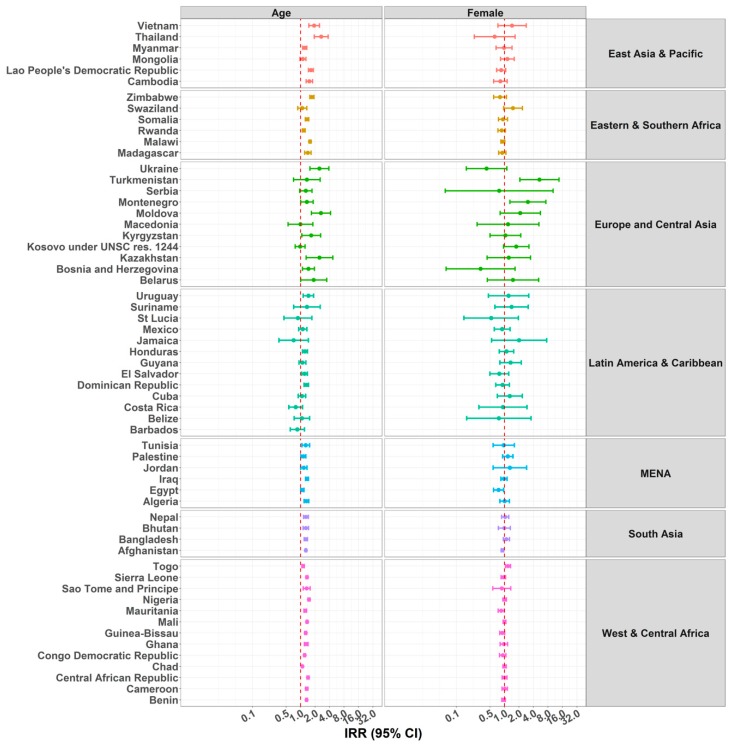
Incidence rate ratios (IRRs) of predictors age and sex for children under five years being home alone. Due to problems of convergence, the estimate of sex for Barbados is not presented.

Figure 3 and Table S5 show the incidence rate ratios (IRR) and 95% confidence intervals (CI) of the number of days the child was home alone in relation to their age and sex. Age was significantly associated with child home alone in 34 countries, meaning children aged three years or older were more likely to be home alone. All countries from EAP (except Mongolia), ESA (except Swaziland), SA, and WCA, had an IRR larger than one, suggesting that older children were more likely to be home alone. Similar significant associations were observed in some ECA countries, namely Ukraine, Moldova, Kazakhstan, and Bosnia and Herzegovina. In LAC, the only countries with a significant IRR larger than one were the Dominican Republic, Honduras, and Uruguay. Finally, in MENA, the same association was observed only for Algeria and Iraq. Overall, a positive association was found between age and child home alone, and the IRR ranged between 0.7 and 2.7.

Regarding sex, no clear pattern of association was observed with child home alone. Compared to boys, girls were significantly less likely to be home alone in Afghanistan, Egypt, Guinea-Bissau, and Mauritania. Conversely, girls were significantly more likely to be home alone in Togo, Serbia, and Turkmenistan.

**Figure 4:**
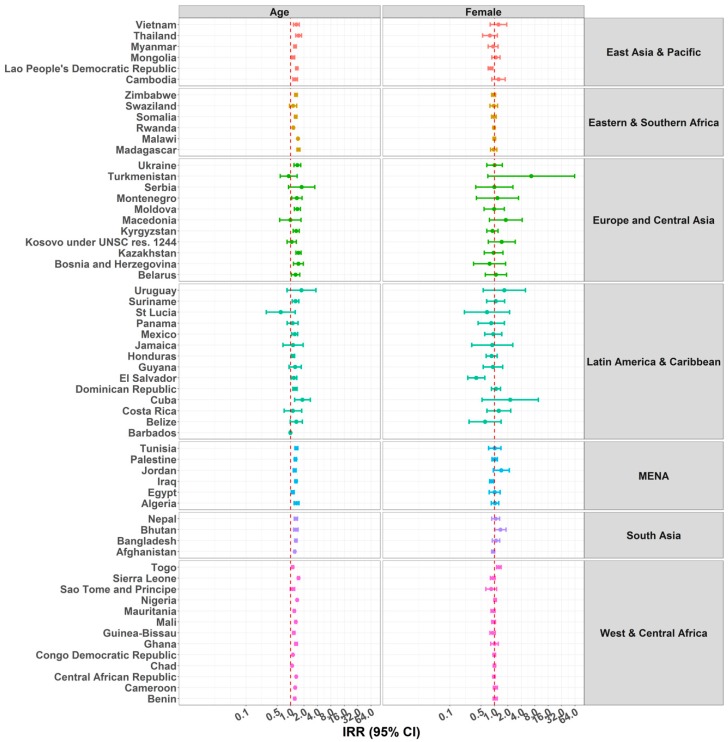
IRRs of predictors age and sex for children under five years under the supervision of another child aged 10 years or younger. Due to problems of convergence, the estimate of sex for Barbados is not presented.

Figure 4 and Table S7a show the IRR of each country’s model for the same predictors but estimating their association with the outcome of child supervised by another child. Age was significantly associated in 41 countries, meaning that children aged three years or older were more likely to be supervised by another child younger than 10 years of age. All countries from EAP, ESA (except Swaziland), MENA, SA, and WCA (except Sao Tome and Principe), had an IRR larger than one, suggesting that older children were more likely to be supervised by another child compared to younger children. The same tendency was observed in some countries in ECA and LAC. More specifically for the former, Ukraine, Montenegro, Moldova, Kyrgyzstan, Kazakhstan, Bosnia and Herzegovina, and Belarus; and for the latter, Suriname, Mexico, Honduras, the Dominican Republic, and Cuba, were included. Overall, a positive association was found between age and child supervised by another child, and the IRR values ranged between 0.60 and 1.84.

The association with child sex was also very weak within countries. Compared to boys, girls were significantly less likely to being supervised by another child in Laos, El Salvador, and Iraq, and in Afghanistan the association was strong but not statistically significant. In contrast, girls were significantly more likely to be supervised by another child in Togo and Turkmenistan.

### 3.4. Mother’s Education

**Figure 5:**
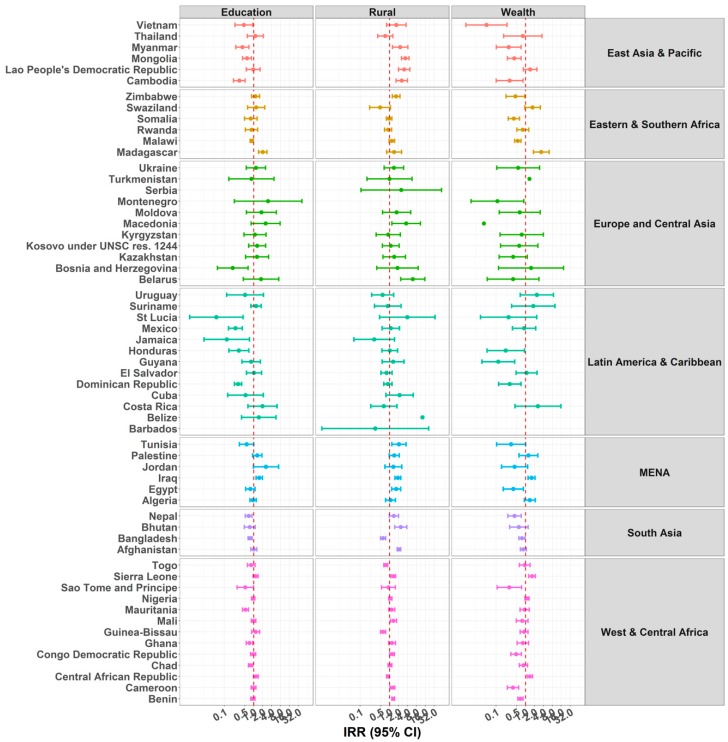
IRRs of predictors education, rurality, and wealth for children under five years old being home alone. Due to problems of convergence, the following countries were excluded from some analyses: Barbados, with the variables mother’s education and wealth; Serbia, with the variables mother’s education and wealth; Montenegro, with the variable rurality; Jamaica, Belize, and Cuba, with variable wealth. In addition, the 95% confidence interval (CI) for the estimates of wealth in Turkmenistan and Macedonia, and for the variable rurality in Belize, were too wide to be represented in the figure.

The IRR of the association between a child home alone and maternal education is shown on Figure 5 and Table S4. In EAP countries, higher maternal education was associated with lower IRR for the number of days the child was home alone in Cambodia, Myanmar, Mongolia, and Vietnam. The same tendency was observed in LAC and SA countries, and the IRR reached the level of significance in the Dominican Republic, Honduras, Jamaica, Mexico, Saint Lucia, Bangladesh, and Nepal. The pattern of association was less consistent in other regions, with both positive and negative associations observed within each region. For instance, higher maternal education was associated with increased IRR for a child home alone in Madagascar and a decreased IRR in Malawi, both in the ESA. The same pattern was observed in MENA with an increased IRR of child home alone in relation to maternal education in Iraq and Jordan, and a decreased IRR in Tunisia. Similar contrasting patterns were observed in WCA. Finally, mother’s education was associated with lower IRR in Bosnia and Herzegovina within ECA.

**Figure 6:**
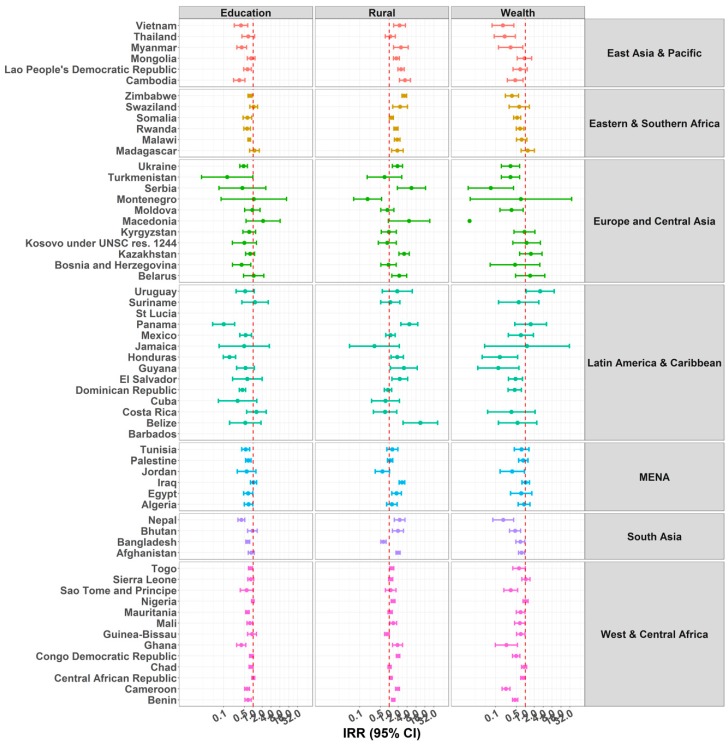
IRRs of predictors Education, Rurality, and Wealth for Children under Five Years under the Supervision of Another Child Aged 10 Years or Younger. Due to problems of convergence, the following countries were excluded from some analyses: Barbados and Saint Lucia, with the variables mother’s education, rurality, and wealth; and Cuba for the variable wealth. In addition, 95% CI for the estimates of wealth in Macedonia were too wide to be represented in the figure.

Estimates for the outcome child supervised by another child were more consistent. Overall, we observed lower IRR of number of days children were supervised by another child in relation to mother’s education. This trend was observed in most of the countries across the six regions and reached the level of significance for 28 countries (Figure 6 and Table S7a).

### 3.5. Rurality and Socio-Economic Status

Children living in rural areas were more likely to be home alone in most EAP (except Thailand), MENA, and SA (except Bangladesh) countries. The same increased likelihood of being home alone in rural areas was observed for Zimbabwe in ESA, and for Belarus and Macedonia in ECA. Results from WCA suggest both positive and negative significant associations. In Central African Republic, Guinea, and Togo, rurality was associated with a significant decrease in the likelihood of being home alone, whereas in Benin, Cameroon, Mali, and Sierra Leone, rurality was associated with a significant increase in being home alone. Finally, in LAC, rurality was only significantly associated with an increase in being home alone in Belize (Figure 5 and Table S4).

For the outcome child supervised by another child, we observed positive associations indicating that children from rural areas were more likely to be supervised by another child in 32 countries. Overall, except for Montenegro, Jordan, Bangladesh, and Guinea Bissau, all remaining associations exhibited the same positive tendency (Figure 6 and Table S7b).

Regarding wealth, we observed both positive and negative associations between wealth quintiles (fifth vs. first quintile) and number of days the child was home alone. For EAP, ECA, LAC, and SA regions, children living in families at the highest wealth quintile were less likely to be home alone compared with children living in families in the lowest wealth quintile. However, in the ESA, MENA, and WCA regions, both positive and negative associations were observed with no clear pattern of associations.

Relative to the number of days a child was home alone, the pattern of associations was more consistent for the outcome child supervised by another child. Most of the countries exhibited an IRR lower than one in relation to wealth scores (fifth vs. first quintile). Only Uruguay exhibited the opposite pattern with an IRR significantly higher than one, suggesting young children are more likely to be supervised by another child in wealthier households.

### 3.6. Number of Individuals in the Household

**Figure 7:**
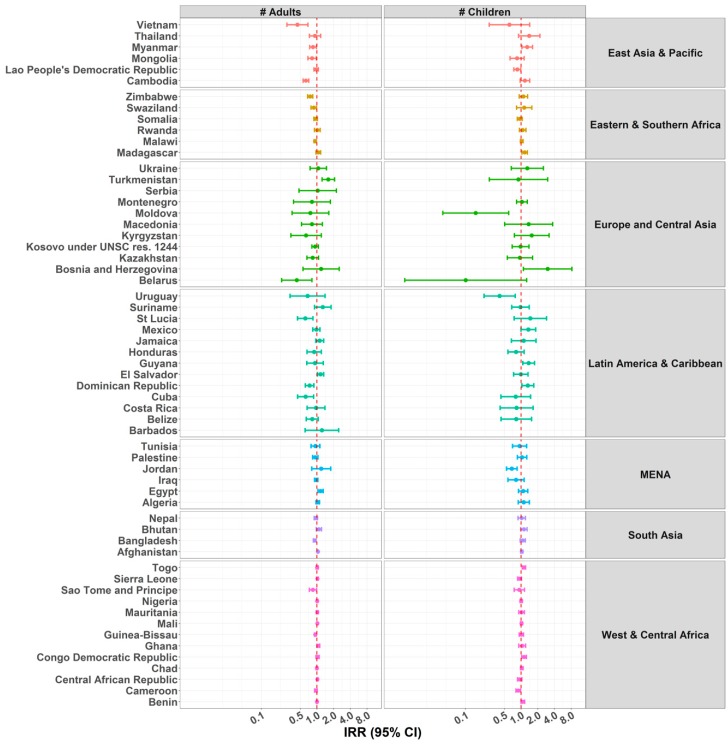
IRRs of predictors Adults and Children Living in the Household for Children under Five Years Home Alone. Due to problems of convergence, Serbia and Barbados were excluded from some analyses for the variable of number of children living in the household.

Overall children living in households with a higher number of adults were either less likely to be home alone, or there was no pattern of association. This was the case across all regions, except in Turkmenistan, El Salvador, Egypt, and Afghanistan, where children were more likely to be home alone with a higher number of adults in the household. There was no clear pattern of association between number of children in the household and number of days the child was home alone, with both positive and negative associations being documented across countries (Figure 7 and Table S4).

**Figure 8:**
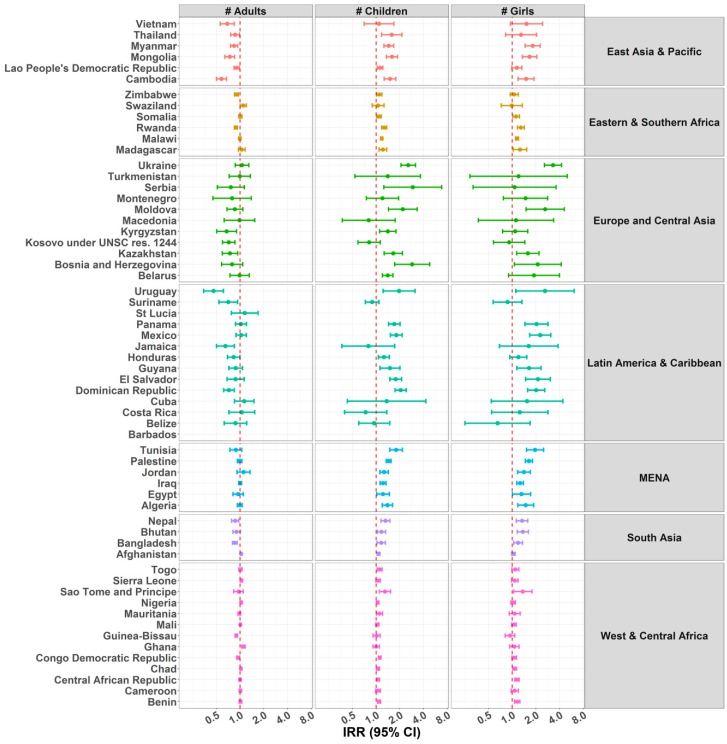
IRRs of predictors Adults and Children Living in the Household for Children under Five Years under the Supervision of Another Child Aged 10 Years or Younger. Due to problems of convergence, the following countries were excluded from some analyses: Barbados, with the variables number of adults, number of children, and number of girls living in the household; and Saint Lucia, with the variables number of children and number of girls living in the household.

For the outcome child supervised by another child, a clear pattern showed that children in households with a higher number of adults were less likely to be supervised by another child, except for Swaziland, Afghanistan, Sierra Leone, and Ghana (Figure 8 and Table S7b). Unlike the results for children under five years home alone displayed in Figure 7, the pattern also showed that children in households with a higher number of children aged 10–14 were more likely to be supervised by another child, with a significant IRR in 43 countries. Finally, children from families with a higher number of girls were overall more likely to be supervised by another child, and this was consistent across the six regions. This positive association reached the level of significance for 30 countries.

## 4. Discussion

### 4.1. Prevalence of Non-Adult Supervision

This study is the first to analyze cross-sectional data on child supervision in LMIC, providing evidence that many young children do not receive optimal supervision around the world. Our analyses reveal ample variation in the worldwide prevalence of children under five years of age being home alone from 0.1% in Serbia to 35.3% in Chad or under the supervision of another child less than 10 years old from 0.2% in Barbados to 50.6% in Central African Republic. Overall, WCA countries displayed the highest numbers in terms of frequency and duration of both types of nonadult supervision. In other regions, high prevalence rates were observed for children home alone (e.g., SA) or under the supervision of another child less than 10 years old (e.g., ESA). Specific countries within these and other regions displayed alarming proportions of children under nonadult supervision. In fact, nonadult supervision appears to be a frequent pattern of care in several countries.

### 4.2. Child Age and Sex

Overall, we observed that an increase in age is associated with an increase in nonadult supervision in LMIC. More specifically, children older than three years were more likely to be home alone or supervised by another child aged nine or younger. Likewise, researchers in HIC have consistently found that children were more likely to stay home alone and to spend larger amounts of time unsupervised as they age. In fact, a child’s age is often the strongest correlate of being home alone [32,33,48]. In the case of children under eight years of age, decreased parental availability tends to be supplemented by care from relatives and nonrelatives rather than children caring for themselves [33]. However, none of these studies included children younger than five years of age [32,48,49,50] nor households headed by minors. A small percentage of child-headed households were included in our sample, although it was under 1% in all countries except Madagascar (1.28%) [51]. Changes in parental supervision practices around late toddlerhood may be partly explained by the end of breastfeeding and the increasing weight and mobility of children, which create a challenge for carrying the child by parents, usually mothers, while walking and working [21]. Distinctive cultural and social norms, particularly in regards to the relative autonomy and expected caregiving role of children, may also explain variations in supervision practices [52].

In our study, we found no strong evidence supporting the role of the child’s sex in practices related to child supervision. Evidence from the U.S. indicated that boys are slightly more likely than girls to be home alone [32,35], which was related to the finding from Canada that “parents communicate to young children in ways that may promote greater risk taking by boys than girls and greater perceived injury vulnerability among girls than boys” [53]. Further, in Bangladesh, Stewart et al. [54] found that girls are considered to need greater protection than boys, and are thus more closely supervised by parents and kept inside the home. It is unclear to what extent the latter findings also apply to children younger than adolescents. Sex differences, as we will see below, remain in other dimensions of child supervision.

### 4.3. Mother’s Education

Parental education may also influence parents’ perception of their children’s ability to supervise or care for themselves and parents’ awareness of risks and available resources. Maternal education seemed protective for children being home alone in SA, EAP, and LAC, and specific countries within other regions (e.g., Malawi, Tunisia, and Bosnia and Herzegovina), and protective for children under the supervision of another child in almost all the countries studied. In some cases, this may be explained by increased access to alternative childcare such as daycare centers and/or private sitters, since women with higher levels of education may hold employment with a better salary and working conditions. In contrast, evidence showed that higher parental education is associated to a higher likelihood of (older) children being home alone in the U.S. [32,35], yet this association does not hold for all age groups [32,36]. In relation to our study, results from Madagascar, Montenegro, Iraq, and Jordan point in this direction. We are uncertain, however, of the mechanisms that explain this association in these countries, particularly because these countries do not share national characteristics that could be advanced as alternative explanations of why children are more likely to be home alone when their mothers have more formal education.

### 4.4. Rurality and Socio-Economic Status

Our findings reveal significant inequities in child supervision. With a handful of exceptions, more children are under the supervision of another child in rural than in urban areas, and a similar trend was observed for home alone in EAP, MENA, and specific countries in other regions. With increases in urbanization and female employment, the demand for alternative childcare has also increased in many LMIC [55], yet availability and cost are major barriers globally. Any access to daycare centers are generally located in urban settings and only accessible to upper income families or those in formal employment [31]. Despite the rapid decrease, 51% of the population in LMIC live in rural areas; the percentage is even higher in SA, ESA, and WCA [56]. In rural areas, non-lactating children generally stay at home while their parents attend to the fields or sell their produce in nearby markets, often carrying their youngest, nursing infant with them. Having multiple caretakers of infants and young toddlers (alloparental care) is common in societies such as the Efé (Pygmies) and the Lese of North-Eastern DRC [57,58]. The widespread practice of shared caregiving, along with close housing arrangements and family and community networks, may assure parents that someone will be available to respond if their children need help. Other times, it may not really be a choice. Rheinländer, et al. [59] found Vietnamese highland ethnic minority children spent more time unsupervised by adults than lowland ethnic minority children; in the absence of alternative childcare solutions, this allows the parents of the former to attend to the fields. Ethnographic studies, including observation of housing arrangements and child care and supervision practices in diverse sub-groups of the population, are needed to assess the ecological validity of these items in different settings, assist with the interpretation of findings, and inform contextually-appropriate interventions to support children and families in each country [60].

In many countries, young children from the poorest households are more likely experience nonadult supervision [61]. Our analyses highlight a global pattern for young children under the care of another child (except Uruguay) and for children home alone in EAP, ECA, LAC, and SA regions. This resonates with other studies documenting the limited ability of parents to pay for or access alternative child care among low income families and those with poor working conditions [31,62]. As of March 2012, none of the countries in our sample except Algeria, Mexico, and Turkmenistan had benefits for childcare or school costs, and means-tested benefits in Tunisia and Serbia were only available to families with incomes below a certain level [63]. In MENA, ESA, and WCA, both positive and negative associations with socioeconomic status coexist for children home alone. Analyses with national data in the U.S. show that being home alone is more common in upper-income families [32,37,48], even if few children aged 5–7 years are home alone regardless of their income level [36] and children from both low- and high-income groups spend about the same amount of time home alone [32]. This may be partly explained by richer families’ higher likelihood to live in neighborhoods perceived as safe [36]. Other researchers, however, did not find a significant association between being home alone and income after controlling for other factors [34]. Disentangling income, education, and other closely-related factors is a difficult task that must nonetheless be undertaken. More within-country analyses are needed as large variations require different policy solutions that can effectively and efficiently respond to the specific combinations of factors that increase the probability that a child younger than five years will be in nonadult care in each location.

### 4.5. Number of Individuals in the Household

Most children in LMIC are generally cared for by adults and older children. In fact, across all regions and with very few exceptions, children living in households with a higher number of adults were less likely to be home alone even if no clear pattern emerged for the frequency of this practice. Children in households with a larger number of adults were less likely to be under the supervision of another child under the age of 10 years, whereas children living with more children aged 10 to 14 years were more likely to be under the supervision of another child. Moreover, a positive and significant association was found between the number of girls aged 10 to 14 years and this type of nonadult supervision. Research on the presence of nonparental adults in the household has produced mixed results in the U.S., since different child age groups were analysed. However, some caution in integrating their conclusions must be taken since none of these studies considered children under five years of age. In the context of LMIC, researchers documented how single parenthood [11] and large family size, particularly for families with limited resources [21,62,64,65], can interfere with child supervision because of the stress placed on parents and family resources. The absence of a social support network (e.g., as a result of migration) can also increase supervision challenges and compel some caregivers to bring children to work or leave them home alone [31,64,65,66]. Ironically, sometimes parents work caring for other families’ children [31]. The fact that children from families with a higher number of girls aged 10 to 14 years were overall more likely to be supervised by another child across the six regions in our study highlights the importance of gender roles and expectations, particularly girls’ contribution to sibling care. Although this may be associated with notions of better supervision competences in girls relative to boys [67,68], literature on sex differences in children has also noted that girls are the subject of discriminatory practices within household dynamics. Moreover, childcare responsibilities may have an impact on supervisors’—mostly girls’—academic performance since less time is available to complete school work. Along this line, Dahlblom et al. [22] raised concerns about the “narrowing of life options” of children with intense caretaking responsibilities in Nicaragua due to lack of basic education and marginalization.

### 4.6. Key Considerations for Future Child Supervision Research

More generally, at least four elements from our analyses should be problematized to help progress the research on child supervision in LMIC. First, “child home alone” and “child supervised by another child” are two different components or ways, perhaps overlapping, of how child supervision is articulated within household dynamics. As such, the two interrelated hypotheses could be advanced. First, since the former is less frequent than the latter, the presence of a second child in the household may facilitate nonadult supervision. Second, adults may use one type of supervision over another because they anticipate child caring to be a relatively simple task that can be easily communicated to other children under the condition that any risks are relatively controlled or because they consider that children are safer by themselves than with other children. However, evidence suggests that parents underestimate supervision needs [69,70,71]. Children under five years of age are typically inquisitive and not able to effectively assess risk, and most children are not ready to handle emergencies until about age 11 or 12, although some children may be mature enough before this age [72]. For young children, constant direct attention in close proximity is recommended [6,73], even if this may need to be adapted to different settings (e.g., level of proximity in agricultural contexts) [3] based on further research [74]. In this line, mothers participating in group discussions in Nepal recommended that “child below five years must not be left alone at all” and that “all the members of family have to look after [the children]” as injury prevention actions [75].

Second, children’s contributions to child care free other household members and thus benefit the family at large. Scholars have documented how children appreciate contributing to their families through caregiving [76] and, with the right conditions, they may build resilience [77,78]. At the same time, even if parents can try to ensure children’s wellbeing in their absence, time spent home alone or under the supervision of another young child has been linked to increases in occurrence and severity of unintentional injuries [29,79] and other negative developmental outcomes [2], particularly among younger children [69,70,71]. More research is needed to assess the extent to which emerging evidence of positive effects linked to child caregiving extends to other dimensions of child development and to younger caregivers. Eliciting children’s experiences directly—both as supervisors and supervisees—according to their capacities is recommended.

Third, at the core of the interpretation of our findings are local understandings of the expression “home alone.” Despite efforts to standardize the translation and administration of questionnaires, these terms inevitably need to be placed in social and geographical context. The concept of “home” may be easily understood to be household or dwelling; this may be further compounded by the need to translate these items into many different languages (Table 1). Conversely, many people live with their extended family in the same dwelling or in adjacent floors, or in compounds or informal settlements. Therefore, the expression “home alone” may not be interpreted as a separate housing unit where children are “really by themselves in an empty house”; rather, there may always be people nearby and children may not be constrained to a closed physical space (i.e., they may be exposed to injuries as pedestrians). In fact, in the context of rapid urbanization, growing slum populations, and displacement and changes to family structure due to war and conflict, crowdedness may seriously challenge privacy and the ability of a child to be completely unnoticed by others. Societies’ conceptions of childhood may also influence how this item is interpreted. For example, some may equate “alone” with “without adult”, yet not necessarily without another child such as a sibling or a domestic worker or live-in aid for child care, many of whom are children themselves [80]. Among the Aaumbo of Namibia, for example, Brown [81] explains how “there is a common saying that ‘children are not people, they are children’. In Aaumbo tradition, if you come to a house and only find that children are home you return and say that you didn’t find anyone home. But it is not to say that children are of little value, quite the contrary.”.

Lastly, any understanding of both consequences and factors associated with child supervision should consider environmental hazards in the house and the community. In exploring these factors, attention must be paid to their association with frequency of use, as more time in nonadult supervision may place children at greater risk, and surveys need to be accompanied with local qualitative work to shed light on actual supervision practices, housing and neighborhood conditions, and socio-cultural norms across diverse groups of the population. Additionally, local availability of alternative quality childcare services and policies and information on safety and supervision of children also need to be documented in each setting. This information is crucial to accurately interpret large survey results and to generate policy and programming solutions that are relevant locally.

### 4.7. Limitations

In addition to the inherent limitations of cross-sectional designs, our analyses were limited to data within countries and not for the combined dataset. Indeed, country datasets could not be pooled due to different sample weighting procedures across countries. Second, the most recent national-level survey data available on January 2018 were used for all countries including the items of interest. Recent MICS 5 and DHS VII surveys for some countries were not included as the databases were not yet publicly available. Third, parental availability for supervision was assumed constant within country, although the data collection fieldwork (typically lasting 2–6 months) may have extended over holidays and labor-intensive periods when adult caregivers may have been differentially available to supervise. Fourth, despite careful translation and testing of the questionnaires and training of enumerators in bias-free interviewing at the country level, the understanding of what constitutes “alone” may vary across cultures and settings (e.g., depending on housing arrangements) and administration of these items face-to-face may have resulted in bias due to inaccurate recall and/or social desirability, likely resulting in an underestimation of these practices. Additionally, in some countries, questionnaires were not formally translated into (all) local languages and were rather translated orally during administration. Other studies have found that the use of home alone is often underreported because of guilt, fear of legal consequences, or problems with recall [82]. The extent to which this applies to contexts where children are expected to assist with childcare from a very young age remains to be determined. Assessing parental perceptions of appropriate care in their particular context in future research on risk factors would thus be useful. Fifth, both MICS and DHS are household surveys, so children not residing in the household (e.g., in institutional care) are not captured in these results, yet may be equally subject to nonadult supervision. Exclusion of other children as a result of national fieldwork challenges also needs to be considered (e.g., access to certain neighborhoods due to violence and social unrest, and denied access to upper income gated communities). Sixth, rural population refers to people living in rural areas as defined by national statistical offices. Because what constitutes a city or metropolitan area varies from one country to another, cross-country comparisons on this variable should be drawn with caution. Seventh, the low prevalence of a child being home alone in some countries interfered with some analyses and resulted in wide confidence intervals. Lastly, some differences in sampling and survey design between MICS and DHS need to be considered when comparing across countries. Most notably, whereas MICS only includes all usual members of the household in the household roster, DHS adds visitors who spent the previous night in the household, thus potentially overestimating the number of adults and children available to care. Respondents for information about children less than five years of age are mothers or primary caregivers of these children living in the household in the case of MICS, whereas only biological mothers are included in DHS. This may have resulted in the exclusion of coverage indicators for orphans and foster children in the DHS surveys, and may also have influenced the probability of providing information for a given child. This inconsistency in sampling is one reason results are comparable within country but not across countries.

## 5. Conclusions

Our study forms an important contribution by providing evidence of nonadult supervision of young children in large nationally representative samples of children in 61 LMIC. We also identified factors that require further study to better understand the determinants of nonadult supervision within countries and across regions. Collecting, analyzing, and disseminating reliable data are first and necessary steps to raise public awareness of this global public health issue. The inclusion of these items in the MICS and DHS surveys is a step in the right direction to inform international comparisons and policy efforts related to child supervision. An ecological approach to child development and protection that considers supervision and multiple other factors (e.g., child temperament and behavior and contextual factors) and connects to children’s rights as formulated in the Convention of the Rights of the Child (1989) is recommended. This is a starting point from which to guide future data collection and policy efforts. Aggregate figures reveal that this is a global issue with consequences for both individual children and their societies. Educational and socio-economic inequalities seem to drive child supervision practices worldwide that often coexist with insufficient support to families. The need for accessible quality alternative childcare supports and policies is urgent to ensure that children are adequately supervised and families are supported in this task.

## Figures and Tables

**Table 1:** Summary characteristics of surveys and prevalence of nonadult supervision practices of child home alone and child supervised by another child by region and country.

Region	Country	Survey	Language(s) of Survey	Children Aged <5 Years at Home Alone				Children Aged <5 Years at Home Supervised by Another Child <10 Years			
				Days/Week (%)			N	Days/Week (%)			N
				0	1≥	3≥		0	1≥	3≥	
East Asia and Pacific (EAP)	Cambodia	DHS VII—2014	Khmer	95.9	4.1	2.2	7004	92.9	7.1	4.3	6991
	Lao People’s Democratic Republic	MICS 4—2012	Lao	94.5	5.5	1.6	10,988	89.2	10.8	5.4	10,807
	Mongolia	MICS 5—2014	Mongolian	95.8	4.2	0.9	6051	91.8	8.3	1.7	6045
	Myanmar	DHS VII—2016	Myanmar	93.3	6.7	3.2	4666	88.6	11.4	5.9	4664
	Thailand	MICS 4—2013	Thai	98.5	1.5	0.4	9703	96.2	3.8	1	9687
	Vietnam	MICS 5—2014	Vietnamese	98.4	1.6	0.6	3317	94	6	2	3318
Eastern and Southern Africa (ESA)	Madagascar	MICS 4—2012	Malagasy	83.2	16.8	11.0	2983	75.6	24.4	16.6	2982
	Malawi	MICS 5—2014	Chichewa; Tumbuka	82.6	17.4	7.8	18,941	66.2	33.8	17.4	18,905
	Rwanda	DHS VII—2015	Kinyarwanda	93	7	3.8	7604	69.4	30.6	20.3	7580
	Somalia	MICS 4—2011	Somali	86.8	13.2	5.9	4674	75.7	24.3	15.1	4607
	Swaziland	MICS 5—2014	siSwati	93.5	6.5	3.2	2675	89.2	10.8	4.9	2679
	Zimbabwe	MICS 5—2014	Shona; Ndebele	95.2	4.8	1.3	9883	83.9	16.1	5.7	9880
Europe and Central Asia (ECA)	Belarus	MICS 4—2012	Russian	99.5	0.6	0.1	3442	96.7	3.3	0.8	3441
	Bosnia and Herzegovina	MICS 4—2012	Bosnian; Serbian; Latin and Cyrillic alphabets	99.4	0.6	0.2	2296	98.6	1.4	0.9	2293
	Kazakhstan	MICS 5—2015	Kazakh; [Russian]	99.3	0.7	0.2	5504	96.4	3.6	0.9	5503
	Kosovo under UNSC res. 1244 ^1^	MICS 5—2014	Albanian; Serbian	95.9	4.1	1.1	1646	96.4	3.7	0.5	1646
	Kyrgyzstan	MICS 5—2014	Kyrgyz; Russian	99.3	0.7	0.2	4564	96.4	3.6	1.3	4559
	Macedonia	MICS 4—2011	Macedonian; Albanian	98.2	1.8	1	1367	96.8	3.2	1.1	1359
	Moldova	MICS 4—2012	Romanian; [Russian]	98.6	1.5	0.3	1868	95	5	1.6	1864
	Montenegro	MICS 5—2013	Montenegrin	99	1	0.4	1417	98	2.1	0.6	1417
	Serbia	MICS 5—2014	Serbian	99.9	0.1	0	2718	99.2	0.9	0.1	2718
	Turkmenistan	MICS 5—2016	Turkmen; Russian	99.2	0.8	0	3618	99.6	0.5	0.1	3757
	Ukraine	MICS 4—2012	Ukranian; Russian	98.4	1.6	0.5	4378	94.0	6.0	2.5	4376
Latin America and Caribbean (LAC)	Argentina	MICS 4—2012	Spanish	94.3	5.7	1.7	34,137	96.1	3.9	0.7	32,727
	Barbados	MICS 4—2012	English	99.1	0.9	0.7	461	99.8	0.2	0.2	461
	Belize	MICS 4—2011	English	99.2	0.8	0.1	1941	98.2	1.8	0.8	1939
	Costa Rica	MICS 4—2011	Spanish	98.2	1.8	0.6	2262	97.7	2.3	1.4	2259
	Cuba	MICS 5—2014	Spanish	97.7	2.3	0.9	5627	99.5	0.6	0.2	5626
	Dominican Republic	MICS 5—2014	Spanish	98	2	0.7	19,835	97.5	2.5	0.9	19,648
	El Salvador	MICS 5—2014	Spanish	97.8	2.2	0.7	7333	98.6	1.4	0.2	7333
	Guyana	MICS 5—2014	English	96.5	3.6	1.3	3355	96.6	3.4	1.2	3351
	Honduras	DHS VI—2012	Spanish	98.1	1.9	0.5	9973	95.8	4.3	1	9961
	Jamaica	MICS 4—2011	English	98.8	1.2	0.4	1638	99.2	0.8	0.4	1632
	Mexico	MICS 5—2015	Spanish	97.2	2.8	0.3	8059	96.3	3.7	0.8	8054
	Panama	MICS 5—2013	Spanish	98.6	1.3	0.1	5797	97.6	2.4	0.6	5794
	St Lucia	MICS 4—2012	English	97.3	2.7	0	292	97.3	2.7	0	292
	Suriname	MICS 4—2010	Dutch	97.4	2.6	0.4	3287	96.1	3.9	0.6	3224
	Uruguay	MICS 4—2013	Spanish	97.3	2.9	1.2	1599	97.6	2.5	0.5	1600
Middle East and North Africa (MENA)	Algeria	MICS 4—2013	French	96.5	3.5	1	14,593	96.9	3.1	1	14,562
	Egypt	DHS VI—2014	Arabic	97.6	2.4	1	15,843	98	2.0	1	15,843
	Iraq	MICS 4—2011	Arabic; Kurdish	95.5	4.5	1.9	36,309	93.7	6.3	3	36,299
	Jordan	DHS VI—2012	Arabic	97.9	2.2	0.5	10,284	93	7	1.4	10,284
	Palestine	MICS 5—2014	Arabic	95.8	4.2	1.1	7816	88.5	11.5	3.3	7813
	Tunisia	MICS 4—2012	Arabic (Tunisian)	93.9	6.1	1.8	2893	89.3	10.7	3.5	2889
South Asia (SA)	Afghanistan	MICS 4—2011	Dari; Pashto	71.5	28.5	11.1	14,442	71.0	29.0	14.9	14,127
	Bangladesh	MICS 5—2013	Bengali; [English]	90.7	9.3	4.7	20,712	92.0	8.0	4.5	20,692
	Bhutan	MICS 4—2010	English (Dzongkha, Lhotshamkha, Sharchopkha)	93.3	6.7	2.7	6241	90.1	9.9	4.1	6236
	Nepal	MICS 5—2014	Nepali; Maithili; Bhojpuri	86.8	13.2	8.2	5333	83.3	16.8	11.1	5307
West and Central Africa (WCA)	Benin	MICS 5—2014	French	72.5	27.6	11.1	12,232	79.1	20.9	7.6	12,234
	Cameroon	MICS 5—2014	French	85.8	14.2	7.6	7015	73.3	26.7	16.0	7004
	Central African Republic	MICS 4—2010	French	77.7	22.3	14.6	10,220	49.5	50.6	37.0	10,025
	Chad	DHS VII—2015	English	64.7	35.3	20.3	10,619	51.7	48.3	31.9	10,405
	Congo Democratic Republic	DHS VI—2014	Kikongo; Lingala; Swahili; Tshiluba	81.1	18.9	11	8025	54.1	45.9	33.5	7890
	Ghana	MICS 4—2011	English	83.5	16.5	10.7	7518	85.8	14.3	9.6	7531
	Guinea-Bissau	MICS 5—2014	Portuguese	78.7	21.3	16.7	7445	80.4	19.6	14.5	7395
	Mali	MICS 5—2015	French (Bamanan; other national languages)	75.2	24.8	12.3	16,100	77.5	22.5	10.1	16,061
	Mauritania	MICS 4—2011	Arabic	89.2	10.8	4.2	8954	82.1	17.9	9.3	8852
	Nigeria ^2^	MICS 4—2011	English	76.1	23.9	12.2	25,044	64.6	35.4	19.2	24,855
	Sao Tome and Principe	MICS 5—2014	Portuguese	92.5	7.5	2.6	2016	86.4	13.6	6.3	1991
	Sierra Leone	MICS 4—2010	English	79.0	21.0	11.3	8342	81.5	18.5	9.9	8290
	Togo	DHS VI—2014	Adja; Akébou; Akposso; Ana-Ifè; Bassar; Ewé; Kabyè; Kotokoli; Lamba; Mina; Moba; Nawdem; Tchokossi	82.8	17.2	10.4	6660	76.6	23.4	13.5	6611

^1^ UNSC res. = United Nations Security Council resolution; ^2^ Questionnaires were not translated into local languages yet, field staff during the pre-test were fluent and competent in local languages and cultures. **Notes:** a. Language(s) used only in pre-test are shown in square brackets. b. Questionnaires were not translated, yet standardized translations were provided to teams administering survey in local languages indicated in (parenthesis).

## Supplementary Materials

The following are available online at http://www.mdpi.com/1660-4601/15/8/1564/s1. Table S1. Stages of modelling outcomes ‘Child home alone’ and ‘Child supervised by another child.’ Table S2. Operationalization of low and high levels of mother’s education per country. Table S3. Statistics per country of number of days child was home alone, and descriptives of sex, age, and mother’s level of education used in the models for number of days child was home alone. Table S4. Descriptives per country of number of children aged 10–14 years living in the household, number of adults aged 15 years and older living in the household, wealth index (percentage), and residence used in the models for number of days child was home alone. Table S5. Predictors of number of days child was home alone (incidence rate ratio). Table S6. Statistics per country of number of days child was supervised by another child, and descriptives of sex, age, and mother’s level of education used in the models for number of days child was supervised by another child. Table S7. Descriptives of number of children aged 10–14 years living in the household, number of girls aged 10–14 years living in the household, number of adults aged 15 years and older living in the household, wealth index (percentage), and residence used in the models for number of days child was supervised by another child. Table S8a. Predictors of number of days child was supervised by another child (Incidence Rate Ratio). Table S8b. Predictors of number of days child was supervised by another child (Incidence Rate Ratio).
