# Supplementary material for: Nonadult Supervision of Children in Low- and Middle-Income Countries: Results from 61 National Population-Based Surveys
Source: Int J Environ Res Public Health. 2018 Jul 24;15(8):1564. doi: 10.3390/ijerph15081564 (PMC6121274; doi:10.3390/ijerph15081564)
Supplement: Supplementary file 1 [file ijerph-15-01564-s001.docx]

**Table S1.** Stages of modelling outcomes ‘Child home alone’ and ‘Child supervised by another child.’

| **Stage** | **Predictor of interest** | **Child home alone** | **Child supervised by another child** |
| --- | --- | --- | --- |
| 1 | Age and Sex | Log (µCLA_ij_) = β_0_ + **β_1_sex_ij_** + **β_2_age_ij_**+ u_j_ | Log (µCSAC_ij_) = β_0_ + **β_1_sex_ij_** + **β_2_age_ij_**+ u_j_ |
| 2 | Rural | Log (µCLA_ij_) = β_0_ + β_1_sex_ij_ + β_2_age_ij_+ **β_3_rural_ij_** + u_j_ | Log (µCSAC_ij_) = β_0_ + β_1_sex_ij_ + β_2_age_ij_+ **β_3_rural_ij_** + u_j_ |
| 3 | Wealth Index | Log (µCLA_ij_) = β_0_ + β_1_sex_ij_ + β_2_age_ij_+ β_3_rural_ij_ + β_4_mother_edu_ij_ + β_5_adults_ij_ + **β_6_socioeconomic_ij_** + u_j_ | Log (µCSAC_ij_) = β_0_ + β_1_sex_ij_ + β_2_age_ij_+ β_3_rural_ij_ + β_4_mother_edu_ij_ + β_5_adults_ij_ + **β_6_socioeconomic_ij_** + u_j_ |
| 4 | Maternal education | Log (µCLA_ij_) = β_0_ + β_1_sex_ij_ + β_2_age_ij_+ β_3_rural_ij_ + **β_4_mother_edu_ij_** + u_j_ | Log (µCSAC_ij_) = β_0_ + β_1_sex_ij_ + β_2_age_ij_+ β_3_rural_ij_ + **β_4_mother_edu_ij_** + u_j_ |
| 5 | Number of adults in household | Log (µCLA_ij_) = β_0_ + β_1_sex_ij_ + β_2_age_ij_+ **β_5_adults_ij_** + u_j_ | Log (µCSAC_ij_) = β_0_ + β_1_sex_ij_ + β_2_age_ij_+ **β_5_adults_ij_** + u_j_ |
| 6 | Number of children in household | Log (µCLA_ij_) = β_0_ + β_1_sex_ij_ + β_2_age_ij_+ β_3_rural_ij_ + β_4_mother_edu_ij_ + β_5_adults_ij_ + β_6_socioeconomic_ij_ + **β_7_kids**_ij_ + u_j_ | Log (µCSAC_ij_) = β_0_ + β_1_sex_ij_ + β_2_age_ij_+ β_3_rural_ij_ + β_4_mother_edu_ij_ + β_5_adults_ij_ + β_6_socioeconomic_ij_ + **β_7_kids**_ij_ + u_j_ |
| 7 | Number of girls in household |  | Log (µCSAC_ij_) = β_0_ + β_1_sex_ij_ + β_2_age_ij_+ β_3_rural_ij_ + **β_8_girls_ij_** + u_j_ |
| Where,  j = child  i = neighborhoods  CLA = child home alone  CSAC = child supervised by another child  sex = sex of the child  age = age of the child in years  rural = residence where the household is located  mother_edu= mother’s education level  socioeconomic = wealth index  adults = individuals aged 15 years and older  children = individuals aged 10 to 14 years  girls = females aged 10 to 14 years  u = random effects | | | |

**Table S2.** Classification of ‘Low’ and ‘High’ Levels of Education per Country

|  | **Country** | **Low Education** | **High Education** |
| --- | --- | --- | --- |
| 1 | Afghanistan | None and Primary | Secondary |
| 2 | Algerie | Sans instruction / Prescolaire /Primaire/Moyen | Secondaire /Superieur |
| 3 | Argentina | Ninguna / Primario | Secundario/ Terciario |
| 4 | Barbados | Primary | Secondary |
| 5 | Belarus | General basic / General secondary | Secondary / Vocational / Higher |
| 6 | Belize | None / Primary | Secondary / CET/ITVET/VOTEC |
| 7 | Bhutan | None / Primary | Secondary |
| 8 | Bosnia | None / Primary | Secondary / Higher |
| 9 | Central African Republic | Aucun / Primaire | Secondaire /Superieur |
| 10 | Chad | Aucun / Primaire | Secondaire /Superieur |
| 11 | Costa Rica | Ninguna Primaria | Secundaria y mas |
| 12 | Democratic Republic of Congo | None / Primary | Secondary + |
| 13 | Ghana | None / Primary / Middle/JSS | Secondary / Higher |
| 14 | Irak | None / Primary | Secondary + / Non-standard curriculum |
| 15 | Jamaica | None / Primary | Secondary / Tertiary |
| 16 | Laos | None / Primary / Lowe secondary | Upper secondary / Post secondary nor tertiatary / Higher |
| 17 | Macedonia | None / Primary | Secondary |
| 18 | Madagascar | Sans instruction /Primaire | Secondaire et + |
| 19 | Mauriatania | Aucun / Coranique/Mahadra/ Primaire | Secondaire et + |
| 20 | Moldova | None and Primary | Secondary / Higher / Professional education |
| 21 | Nigeria | None and Primary | Secondary + |
| 22 | Saint Lucia | None and Primary | Secondary + |
| 23 | Sierra Leona | None and Primary | Secondary + |
| 24 | Somalia | None and Primary | Secondary + |
| 25 | Suriname | None and Primary | Secondary + / Other/Non standard |
| 26 | Thailand | None and Primary | Secondary / Higher |
| 27 | Togo | Aucun / Primaire | Secondaire et + |
| 28 | Tunisia | Aucun / Primaire et assimils | Secondaire et assimils / Superieur |
| 29 | Ukraine | Primary and Secondary | Higher |
| 30 | Uruguay | Hasta primaria / Secundaria | Terciaria |
| 31 | Bangladesh | None / Primary incomplete / Primary complete / Secondary incomplete | Secondary complete or higher |
| 32 | Benin | None and Primary | Secondary 1 / Secondary 2 or Higher |
| 33 | Cameroon | Aucun / Primaire | Secondaire /Superieur |
| 34 | Cuba | Primaria o ninguna | Secundaira / Superior |
| 35 | Domincan Republic | Ninguna / Primaria | Secundaira y Obrero Calficado / Pre TC / Superior |
| 36 | El Salvador | Sin escolarizacion / Primaria | Secundaria / Bachillerato / Universitaria |
| 37 | Guinea Bissau | Nehum / Primario | Secundario e mais |
| 38 | Guyana | None / Primary | Secondary / Higher |
| 39 | Kazakhstan | None / Primary / Lower Secondary | Upper secondary / Technical and Professional / Higher |
| 40 | Kosovo | None / Primary / Lower Secondary | Upper secondary / Higher |
| 41 | Kyrgyzstan | None / Primary / Basic Secondary | Complete secondary / Professional primary-middle / Higher |
| 42 | Malawi | None / Primary | Secondary / Higher |
| 43 | Mali | None | Primary / Secondary 1 / Secondary 2 or Higher |
| 44 | Mexico | Ninguno / Primaria | Secundaria / Media Superior / Superior |
| 45 | Mongolia | None / Primary / Basic | Upper secondary/ Vocational / College / University |
| 46 | Montenegro | None / Primary | Secondary / Higher |
| 47 | Nepal | None / Primary | Secondary / Higher |
| 48 | Palestine | None / Basic | Secondary / Higher |
| 49 | Panama | Ninguna / Primaria | Secundaria / Superior |
| 50 | Sao Tome & Principe | Nehum / Ensino Primario | Ensino secundario / Ensino superior |
| 51 | Serbia | None / Primary | Secondary / Higher |
| 52 | Swaziland | None / Primary | Secondary / Higher / Tertiary |
| 53 | Turkmenistan | None / Primary / Secondary | Primay vocational / Secondary vocational / Higher |
| 54 | Viet Nam | None / Primary / Lower Secondary | Upper secondary / Tertiary |
| 55 | Zimbabwe | None / Primary | Secondary / Higher |
| 56 | Cambodia | No education / Primary | Secondary / Higher |
| 57 | Egypt | No education / Primary | Secondary / Higher |
| 58 | Honduras | No education / Primary | Secondary / Higher |
| 59 | Jordan | No education / Primary | Secondary / Higher |
| 60 | Mynamar | No education / Primary | Secondary / Higher |
| 61 | Rwanda | No education / Primary | Secondary / Higher |

**Table S3.** Statistics per country of ‘Number of days child was home alone,’ and descriptives of ‘Sex,’ ‘Age’ and ‘Mother’s level of education’ used in the models for ‘Number of days child was home alone.’

| **Country** | **Survey** | **Number of days child was home alone** | | | | | | | | **Sex** | | **Age in years** | | | | **Mother's level of education (percentage)** | | ***N*** |
| --- | --- | --- | --- | --- | --- | --- | --- | --- | --- | --- | --- | --- | --- | --- | --- | --- | --- | --- |
|  |  | **0** | **1** | **2** | **3** | **4** | **5** | **6** | **7** | **Male** | **Female** | **Mean** | **SD** | **Min** | **Max** | **1** | **2** |  |
| Afghanistan | MICS4 | 71.5% | 6.7% | 10.7% | 4.8% | 2.6% | 1.2% | 1.3% | 1.2% | 51.1% | 48.9% | 3.11 | 1.39 | 1 | 5 | 94.3% | 5.7% | 14442 |
| Algeria | MICS4 | 96.5% | 1.0% | 1.5% | 0.4% | 0.2% | 0.2% | 0.1% | 0.1% | 51.8% | 48.2% | 2.86 | 1.41 | 1 | 5 | 69.2% | 30.8% | 14593 |
| Bangladesh | MICS5 | 90.7% | 2.5% | 2.2% | 1.5% | 0.8% | 0.7% | 0.7% | 0.8% | 51.3% | 48.7% | 2.99 | 1.42 | 1 | 5 | 39.7% | 60.3% | 20712 |
| Barbados | MICS4 | 99.1% | 0.2% |  | 0.2% |  | 0.4% |  |  | 55.1% | 44.9% | 3.13 | 1.43 | 1 | 5 | 100.0% |  | 461 |
| Belarus | MICS4 | 99.4% | 0.3% | 0.1% | 0.0% |  |  |  | 0.0% | 51.4% | 48.6% | 2.98 | 1.41 | 1 | 5 | 18.9% | 81.1% | 3442 |
| Belize | MICS4 | 99.2% | 0.5% | 0.2% | 0.1% |  |  |  |  | 51.2% | 48.8% | 2.98 | 1.40 | 1 | 5 | 56.6% | 43.4% | 1941 |
| Benin | MICS5 | 72.4% | 6.5% | 9.9% | 3.3% | 2.0% | 1.6% | 1.3% | 3.0% | 49.3% | 50.7% | 2.93 | 1.42 | 1 | 5 | 83.8% | 16.2% | 12232 |
| Bhutan | MICS4 | 93.3% | 2.2% | 1.8% | 0.9% | 0.3% | 0.6% | 0.5% | 0.4% | 51.4% | 48.6% | 2.90 | 1.38 | 1 | 5 | 80.6% | 19.4% | 6241 |
| Bosnia and Herzegovina | MICS4 | 99.4% | 0.3% | 0.1% |  |  | 0.1% | 0.1% | 0.0% | 49.2% | 50.8% | 3.18 | 1.31 | 1 | 5 | 22.8% | 77.2% | 2296 |
| Cambodia | DHS VII | 95.9% | 0.8% | 1.1% | 0.7% | 0.3% | 0.3% | 0.1% | 0.7% | 50.3% | 49.7% | 3.62 | 1.53 | 1 | 5 | 63.9% | 36.1% | 7004 |
| Cameroon | MICS5 | 85.8% | 3.0% | 3.6% | 3.0% | 1.0% | 0.9% | 1.2% | 1.5% | 50.9% | 49.1% | 2.91 | 1.40 | 1 | 5 | 63.3% | 36.7% | 7015 |
| Central African Republic | MICS4 | 77.7% | 2.9% | 4.8% | 3.2% | 2.0% | 2.6% | 3.4% | 3.3% | 49.2% | 50.8% | 2.77 | 1.40 | 1 | 5 | 88.6% | 11.4% | 10220 |
| Chad | DHS VII | 64.7% | 5.2% | 9.9% | 7.5% | 4.4% | 3.7% | 2.1% | 2.5% | 50.6% | 49.4% | 3.10 | 1.49 | 1 | 5 | 91.9% | 8.1% | 10619 |
| Congo Democratic Republic | DHS VI | 81.1% | 3.3% | 4.7% | 3.9% | 1.8% | 1.5% | 2.2% | 1.5% | 49.4% | 50.6% | 1.91 | 1.41 | 1 | 5 | 65.9% | 34.1% | 8025 |
| Costa Rica | MICS4 | 98.2% | 1.1% | 0.1% | 0.2% | 0.0% | 0.3% | 0.0% |  | 50.4% | 49.6% | 2.91 | 1.40 | 1 | 5 | 39.4% | 60.6% | 2262 |
| Cuba | MICS5 | 97.7% | 0.9% | 0.5% | 0.3% | 0.1% | 0.2% | 0.1% | 0.1% | 50.3% | 49.7% | 2.97 | 1.37 | 1 | 5 | 20.4% | 79.6% | 5627 |
| Dominican Republic | MICS5 | 98.0% | 0.9% | 0.4% | 0.3% | 0.1% | 0.1% | 0.1% | 0.1% | 50.8% | 49.2% | 2.95 | 1.41 | 1 | 5 | 36.4% | 63.6% | 19835 |
| Egypt | DHS VI | 97.6% | 0.6% | 0.7% | 0.3% | 0.2% | 0.1% | 0.1% | 0.3% | 52.0% | 48.0% | 1.89 | 1.40 | 1 | 5 | 25.1% | 74.9% | 15843 |
| El Salvador | MICS5 | 97.8% | 1.1% | 0.3% | 0.1% | 0.0% | 0.4% | 0.2% | 0.1% | 50.7% | 49.3% | 3.03 | 1.41 | 1 | 5 | 39.8% | 60.2% | 7333 |
| Ghana | MICS4 | 83.5% | 2.7% | 3.1% | 2.8% | 2.3% | 2.2% | 1.0% | 2.4% | 51.1% | 48.9% | 2.95 | 1.41 | 1 | 5 | 76.2% | 23.8% | 7518 |
| Guinea-Bissau | MICS5 | 78.7% | 2.3% | 2.3% | 2.6% | 2.2% | 4.0% | 2.5% | 5.4% | 50.5% | 49.5% | 2.93 | 1.42 | 1 | 5 | 88.4% | 11.6% | 7445 |
| Guyana | MICS5 | 96.5% | 1.1% | 1.1% | 0.4% | 0.3% | 0.3% | 0.2% | 0.2% | 50.5% | 49.5% | 2.95 | 1.39 | 1 | 5 | 19.9% | 80.1% | 3355 |
| Honduras | DHS VI | 98.1% | 1.1% | 0.3% | 0.2% | 0.1% | 0.0% | 0.2% |  | 52.0% | 48.0% | 2.91 | 1.44 | 1 | 5 | 69.2% | 30.8% | 9973 |
| Iraq | MICS4 | 95.5% | 1.2% | 1.4% | 0.7% | 0.3% | 0.4% | 0.1% | 0.3% | 51.1% | 48.9% | 2.88 | 1.40 | 1 | 5 | 74.8% | 25.2% | 36309 |
| Jamaica | MICS4 | 98.8% | 0.4% | 0.4% | 0.1% | 0.1% |  | 0.1% | 0.1% | 52.5% | 47.5% | 2.99 | 1.46 | 1 | 5 | 79.3% | 20.7% | 1638 |
| Jordan | DHS VI | 97.9% | 1.0% | 0.6% | 0.2% |  | 0.2% |  | 0.1% | 52.1% | 47.9% | 3.70 | 1.48 | 1 | 5 | 10.0% | 90.0% | 10284 |
| Kazakhstan | MICS5 | 99.3% | 0.4% | 0.1% | 0.0% | 0.0% | 0.1% |  |  | 51.4% | 48.6% | 2.97 | 1.41 | 1 | 5 | 26.7% | 73.3% | 5504 |
| Kosovo under UNSC res. 1244 | MICS5 | 95.9% | 1.6% | 1.4% | 0.4% | 0.2% | 0.5% |  |  | 53.0% | 47.0% | 3.00 | 1.41 | 1 | 5 | 53.8% | 46.2% | 1646 |
| Kyrgyzstan | MICS5 | 99.3% | 0.4% | 0.2% | 0.1% | 0.0% | 0.0% |  |  | 51.2% | 48.8% | 2.93 | 1.43 | 1 | 5 | 57.2% | 42.8% | 4564 |
| Lao People's Democratic Republic | MICS4 | 94.5% | 2.1% | 1.9% | 0.6% | 0.3% | 0.3% | 0.2% | 0.2% | 50.7% | 49.3% | 2.91 | 1.42 | 1 | 5 | 76.0% | 24.0% | 10988 |
| Macedonia | MICS4 | 98.2% | 0.1% | 0.7% | 0.3% |  | 0.6% |  | 0.1% | 51.4% | 48.6% | 3.01 | 1.41 | 1 | 5 | 77.6% | 22.4% | 1367 |
| Madagascar | MICS4 | 83.2% | 2.5% | 3.3% | 3.9% | 1.2% | 1.3% | 0.8% | 3.7% | 50.3% | 49.7% | 2.85 | 1.41 | 1 | 5 | 89.9% | 10.1% | 2983 |
| Malawi | MICS5 | 82.6% | 4.9% | 4.7% | 3.2% | 1.5% | 1.1% | 0.6% | 1.4% | 50.0% | 50.0% | 3.00 | 1.39 | 1 | 5 | 83.5% | 16.5% | 18941 |
| Mali | MICS5 | 75.2% | 4.2% | 8.3% | 2.8% | 1.9% | 1.7% | 1.0% | 5.0% | 51.3% | 48.7% | 2.92 | 1.39 | 1 | 5 | 88.6% | 11.4% | 16100 |
| Mauritania | MICS4 | 89.2% | 3.1% | 3.5% | 1.7% | 0.8% | 0.6% | 0.5% | 0.6% | 50.9% | 49.1% | 2.92 | 1.41 | 1 | 5 | 60.1% | 39.9% | 8954 |
| Mexico | MICS5 | 97.2% | 1.7% | 0.8% | 0.1% | 0.1% | 0.1% |  | 0.0% | 50.5% | 49.5% | 3.01 | 1.42 | 1 | 5 | 22.9% | 77.1% | 8059 |
| Moldova | MICS4 | 98.6% | 0.5% | 0.7% | 0.2% | 0.1% |  |  |  | 51.9% | 48.1% | 2.89 | 1.41 | 1 | 5 | 41.6% | 58.4% | 1868 |
| Mongolia | MICS5 | 95.8% | 2.0% | 1.3% | 0.4% | 0.1% | 0.1% | 0.0% | 0.3% | 51.2% | 48.8% | 2.90 | 1.41 | 1 | 5 | 13.2% | 86.8% | 6051 |
| Montenegro | MICS5 | 99.0% | 0.5% | 0.1% | 0.3% |  | 0.1% |  |  | 52.5% | 47.5% | 3.02 | 1.39 | 1 | 5 | 16.0% | 84.0% | 1417 |
| Myanmar | DHS VII | 93.3% | 2.4% | 1.2% | 1.2% | 0.3% | 0.5% |  | 1.1% | 52.3% | 47.7% | 2.96 | 1.43 | 1 | 5 | 62.1% | 37.9% | 4666 |
| Nepal | MICS5 | 86.8% | 2.1% | 2.9% | 2.5% | 0.9% | 1.0% | 0.6% | 3.2% | 52.4% | 47.6% | 3.01 | 1.41 | 1 | 5 | 61.7% | 38.3% | 5333 |
| Nigeria | MICS4 | 76.1% | 4.2% | 7.4% | 4.9% | 2.3% | 2.3% | 1.2% | 1.5% | 51.1% | 48.9% | 2.90 | 1.43 | 1 | 5 | 68.9% | 31.1% | 25044 |
| Palestine | MICS5 | 95.8% | 2.0% | 1.0% | 0.5% | 0.3% | 0.2% | 0.1% | 0.1% | 52.1% | 47.9% | 3.02 | 1.40 | 1 | 5 | 30.4% | 69.6% | 7816 |
| Rwanda | DHS VII | 93.0% | 1.6% | 1.7% | 1.3% | 0.4% | 0.8% | 0.7% | 0.6% | 50.7% | 49.3% | 3.97 | 1.44 | 1 | 5 | 86.3% | 13.7% | 7604 |
| Sao Tome and Principe | MICS5 | 92.5% | 2.6% | 2.3% | 0.5% | 0.3% | 1.0% | 0.3% | 0.3% | 50.7% | 49.3% | 3.07 | 1.40 | 1 | 5 | 70.1% | 29.9% | 2016 |
| Serbia | MICS5 | 99.9% |  | 0.0% | 0.0% |  |  |  |  | 50.3% | 49.7% | 3.13 | 1.41 | 1 | 5 | 63.4% | 36.6% | 2718 |
| Sierra Leone | MICS4 | 79.0% | 3.3% | 6.3% | 3.6% | 2.6% | 1.6% | 0.8% | 2.7% | 49.7% | 50.3% | 2.95 | 1.43 | 1 | 5 | 85.3% | 14.7% | 8342 |
| Somalia | MICS4 | 86.8% | 3.1% | 4.2% | 2.7% | 1.0% | 0.6% | 0.4% | 1.2% | 50.6% | 49.4% | 3.00 | 1.40 | 1 | 5 | 93.7% | 6.3% | 4674 |
| St Lucia | MICS4 | 97.3% | 2.4% | 0.3% |  |  |  |  |  | 49.7% | 50.3% | 2.96 | 1.40 | 1 | 5 | 25.0% | 75.0% | 292 |
| Suriname | MICS4 | 97.4% | 1.9% | 0.3% | 0.1% | 0.0% | 0.1% | 0.1% | 0.1% | 49.7% | 50.3% | 2.90 | 1.39 | 1 | 5 | 57.0% | 43.0% | 3287 |
| Swaziland | MICS5 | 93.5% | 2.2% | 1.1% | 1.3% | 0.6% | 0.5% | 0.2% | 0.5% | 51.1% | 48.9% | 2.94 | 1.38 | 1 | 5 | 41.8% | 58.2% | 2675 |
| Thailand | MICS4 | 98.5% | 0.7% | 0.3% | 0.2% | 0.0% | 0.1% | 0.0% | 0.2% | 51.0% | 49.0% | 3.15 | 1.36 | 1 | 5 | 38.6% | 61.4% | 9703 |
| Togo | DHS VI | 82.8% | 2.2% | 4.6% | 3.8% | 2.0% | 2.0% | 1.1% | 1.5% | 50.4% | 49.6% | 3.96 | 1.45 | 1 | 5 | 80.0% | 20.0% | 6660 |
| Tunisia | MICS4 | 93.9% | 2.7% | 1.5% | 0.6% | 0.5% | 0.3% | 0.2% | 0.2% | 53.3% | 46.7% | 2.92 | 1.40 | 1 | 5 | 50.2% | 49.8% | 2893 |
| Turkmenistan | MICS5 | 99.2% | 0.5% | 0.3% |  |  |  |  |  | 52.5% | 47.5% | 2.96 | 1.40 | 1 | 5 | 88.7% | 11.3% | 3618 |
| Ukraine | MICS4 | 98.4% | 0.7% | 0.4% | 0.1% | 0.0% | 0.2% | 0.1% |  | 50.9% | 49.1% | 3.11 | 1.37 | 1 | 5 | 35.7% | 64.3% | 4378 |
| Uruguay | MICS4 | 97.3% | 0.5% | 1.3% | 0.4% | 0.3% | 0.1% | 0.3% | 0.3% | 52.2% | 47.8% | 3.21 | 1.33 | 1 | 5 | 21.2% | 78.8% | 1599 |
| Vietnam | MICS5 | 98.4% | 0.6% | 0.4% | 0.2% | 0.1% | 0.0% | 0.1% | 0.2% | 51.6% | 48.4% | 2.80 | 1.41 | 1 | 5 | 23.1% | 76.9% | 3317 |
| Zimbabwe | MICS5 | 95.2% | 2.5% | 1.0% | 0.6% | 0.1% | 0.2% | 0.1% | 0.3% | 50.0% | 50.0% | 2.96 | 1.37 | 1 | 5 | 38.9% | 61.1% | 9883 |

**Table S4.** Descriptives per country of ‘Number of children aged 10 - 14 years living in the household,’ Number of adults aged 15 years and older living in the household,’ ‘Wealth index (percentage),’ and ‘Residence’ used in the models for ‘Number of days child was home alone.’

| **Country** | **Survey** | **Number of children aged 10 – 14 years living in the household** | | | | **Number of adults aged 15 years and older living in the household** | | | | | **Wealth index (percentage)** | | | | | **Residence** | | ***N*** |
| --- | --- | --- | --- | --- | --- | --- | --- | --- | --- | --- | --- | --- | --- | --- | --- | --- | --- | --- |
|  |  | **Mean** | **SD** | **Min** | **Max** | | **Mean** | **SD** | **Min** | **Max** | **1** | **2** | **3** | **4** | **5** | **Urban** | **Rural** |  |
| Afghanistan | MICS4 | 1.06 | 1.13 | 0 | 6 | | 4.45 | 2.62 | 1 | 23 | 18.8% | 20.0% | 19.2% | 19.8% | 22.2% | 24.1% | 75.9% | 14442 |
| Algeria | MICS4 | 0.47 | 0.74 | 0 | 4 | | 3.49 | 2.17 | 1 | 16 | 23.6% | 20.5% | 20.6% | 19.5% | 15.8% | 64.6% | 35.4% | 14593 |
| Bangladesh | MICS5 | 0.46 | 0.70 | 0 | 6 | | 3.14 | 1.68 | 1 | 16 | 30.1% | 21.7% | 18.1% | 16.2% | 13.9% | 15.9% | 84.1% | 20712 |
| Barbados | MICS4 |  |  |  |  | | 3.07 | 1.58 | 1 | 11 |  |  |  |  |  | 59.2% | 40.8% | 461 |
| Belarus | MICS4 | 0.18 | 0.43 | 0 | 3 | | 2.33 | 0.82 | 1 | 8 | 14.6% | 19.2% | 17.9% | 21.4% | 26.8% | 72.0% | 28.0% | 3442 |
| Belize | MICS4 | 0.61 | 0.89 | 0 | 4 | | 2.93 | 1.54 | 1 | 11 | 29.6% | 22.6% | 20.0% | 16.1% | 11.8% | 35.1% | 64.9% | 1941 |
| Benin | MICS5 | 0.88 | 1.16 | 0 | 15 | | 3.20 | 2.17 | 1 | 29 | 19.1% | 18.4% | 18.5% | 19.5% | 24.5% | 50.3% | 49.7% | 12232 |
| Bhutan | MICS4 | 0.50 | 0.74 | 0 | 4 | | 3.53 | 1.68 | 1 | 14 | 21.8% | 21.5% | 20.6% | 20.7% | 15.4% | 21.8% | 78.2% | 6241 |
| Bosnia and Herzegovina | MICS4 | 0.22 | 0.50 | 0 | 4 | | 2.91 | 1.21 | 1 | 8 | 17.3% | 20.2% | 21.0% | 18.6% | 22.9% | 34.9% | 65.1% | 2296 |
| Cambodia | DHS VII | 0.41 | 0.69 | 0 | 4 | | 3.30 | 1.74 | 1 | 14 | 24.0% | 18.7% | 15.9% | 17.4% | 23.9% | 26.9% | 73.1% | 7004 |
| Cameroon | MICS5 | 0.84 | 1.07 | 0 | 8 | | 3.21 | 1.81 | 1 | 16 | 17.3% | 23.2% | 22.9% | 20.2% | 16.4% | 45.8% | 54.2% | 7015 |
| Central African Republic | MICS4 | 0.67 | 0.93 | 0 | 10 | | 2.75 | 1.46 | 1 | 13 | 22.2% | 24.9% | 24.2% | 17.9% | 10.7% | 32.2% | 67.8% | 10220 |
| Chad | DHS VII | 0.92 | 1.15 | 0 | 8 | | 2.71 | 1.54 | 1 | 17 | 20.0% | 20.1% | 21.0% | 22.2% | 16.7% | 20.2% | 79.8% | 10619 |
| Congo Democratic Republic | DHS VI | 0.82 | 0.97 | 0 | 6 | | 2.72 | 1.37 | 0 | 15 | 26.2% | 22.2% | 21.2% | 17.1% | 13.4% | 29.7% | 70.3% | 8025 |
| Costa Rica | MICS4 | 0.40 | 0.66 | 0 | 4 | | 2.82 | 1.40 | 1 | 16 | 35.4% | 24.4% | 16.5% | 13.9% | 9.9% | 48.2% | 51.8% | 2262 |
| Cuba | MICS5 | 0.18 | 0.42 | 0 | 3 | | 2.94 | 1.28 | 1 | 11 |  |  |  |  |  | 75.9% | 24.1% | 5627 |
| Dominican Republic | MICS5 | 0.35 | 0.64 | 0 | 5 | | 2.55 | 1.21 | 1 | 10 | 31.1% | 22.8% | 18.5% | 15.1% | 12.6% | 64.4% | 35.6% | 19835 |
| Egypt | DHS VI | 0.37 | 0.71 | 0 | 5 | | 2.64 | 1.49 | 1 | 16 | 18.4% | 18.7% | 20.6% | 20.8% | 21.5% | 40.9% | 59.1% | 15843 |
| El Salvador | MICS5 | 0.42 | 0.70 | 0 | 6 | | 2.92 | 1.43 | 1 | 11 | 23.7% | 21.1% | 20.2% | 18.3% | 16.7% | 56.2% | 43.8% | 7333 |
| Ghana | MICS4 | 0.91 | 1.06 | 0 | 8 | | 2.99 | 1.60 | 1 | 14 | 46.6% | 19.9% | 13.9% | 11.1% | 8.5% | 28.1% | 71.9% | 7518 |
| Guinea-Bissau | MICS5 | 1.13 | 1.22 | 0 | 9 | | 5.04 | 3.01 | 1 | 24 | 30.5% | 24.6% | 22.8% | 14.1% | 8.0% | 25.8% | 74.2% | 7445 |
| Guyana | MICS5 | 0.53 | 0.80 | 0 | 4 | | 3.05 | 1.64 | 1 | 13 | 37.8% | 19.1% | 15.6% | 13.6% | 13.9% | 20.6% | 79.4% | 3355 |
| Honduras | DHS VI | 0.68 | 0.90 | 0 | 7 | | 3.09 | 1.61 | 0 | 13 | 33.0% | 23.2% | 17.8% | 15.3% | 10.7% | 33.7% | 66.3% | 9973 |
| Iraq | MICS4 | 0.83 | 1.14 | 0 | 8 | | 4.13 | 2.79 | 1 | 23 | 34.2% | 23.9% | 18.4% | 13.6% | 9.8% | 54.1% | 45.9% | 36309 |
| Jamaica | MICS4 | 0.55 | 0.79 | 0 | 5 | | 3.08 | 1.68 | 1 | 12 | 23.6% | 23.1% | 21.4% | 17.5% | 14.3% | 59.6% | 40.4% | 1638 |
| Jordan | DHS VI | 0.58 | 0.91 | 0 | 6 | | 2.68 | 1.43 | 1 | 14 | 27.0% | 26.7% | 22.6% | 16.3% | 7.5% | 69.0% | 31.0% | 10284 |
| Kazakhstan | MICS5 | 0.31 | 0.59 | 0 | 3 | | 3.17 | 1.43 | 1 | 12 | 19.5% | 18.9% | 22.3% | 19.8% | 19.5% | 55.2% | 44.8% | 5504 |
| Kosovo under UNSC res. 1244 | MICS5 | 0.50 | 0.92 | 0 | 6 | | 4.74 | 2.45 | 1 | 20 | 24.2% | 19.2% | 19.7% | 19.3% | 17.7% | 35.8% | 64.2% | 1646 |
| Kyrgyzstan | MICS5 | 0.38 | 0.66 | 0 | 4 | | 3.30 | 1.41 | 1 | 9 | 24.3% | 22.1% | 21.2% | 18.2% | 14.3% | 32.9% | 67.1% | 4564 |
| Lao People's Democratic Republic | MICS4 | 0.67 | 0.90 | 0 | 7 | | 3.57 | 1.82 | 1 | 15 | 32.9% | 22.5% | 18.1% | 14.8% | 11.8% | 18.9% | 81.1% | 10988 |
| Macedonia | MICS4 | 0.17 | 0.47 | 0 | 3 | | 3.58 | 1.68 | 1 | 17 | 18.1% | 19.9% | 21.4% | 19.5% | 21.1% | 54.6% | 45.4% | 1367 |
| Madagascar | MICS4 | 0.82 | 1.02 | 0 | 7 | | 2.72 | 1.56 | 1 | 17 | 25.5% | 20.3% | 22.3% | 20.4% | 11.5% | 4.6% | 95.4% | 2983 |
| Malawi | MICS5 | 0.70 | 0.86 | 0 | 8 | | 2.43 | 1.07 | 1 | 10 | 22.6% | 22.1% | 21.5% | 18.7% | 15.2% | 11.2% | 88.8% | 18941 |
| Mali | MICS5 | 1.68 | 1.82 | 0 | 14 | | 5.92 | 4.61 | 1 | 35 | 21.4% | 20.5% | 19.6% | 20.2% | 18.3% | 21.3% | 78.7% | 16100 |
| Mauritania | MICS4 | 0.87 | 1.02 | 0 | 7 | | 3.45 | 2.22 | 1 | 26 | 24.2% | 23.2% | 18.9% | 17.4% | 16.4% | 34.8% | 65.2% | 8954 |
| Mexico | MICS5 | 0.36 | 0.65 | 0 | 4 | | 2.82 | 1.38 | 1 | 11 | 29.4% | 25.9% | 20.0% | 15.7% | 9.1% | 64.1% | 35.9% | 8059 |
| Moldova | MICS4 | 0.20 | 0.47 | 0 | 3 | | 2.37 | 0.94 | 1 | 7 | 13.2% | 17.0% | 16.8% | 18.9% | 34.2% | 55.1% | 44.9% | 1868 |
| Mongolia | MICS5 | 0.32 | 0.58 | 0 | 4 | | 2.51 | 1.01 | 1 | 13 | 24.1% | 20.7% | 19.1% | 17.3% | 18.9% | 58.1% | 41.9% | 6051 |
| Montenegro | MICS5 | 0.23 | 0.56 | 0 | 3 | | 2.87 | 1.31 | 1 | 9 | 18.9% | 20.3% | 18.7% | 20.5% | 21.6% | 63.3% | 36.7% | 1417 |
| Myanmar | DHS VII | 0.54 | 0.77 | 0 | 5 | | 3.24 | 1.72 | 1 | 17 | 30.4% | 22.7% | 17.9% | 16.6% | 12.3% | 21.1% | 78.9% | 4666 |
| Nepal | MICS5 | 0.50 | 0.80 | 0 | 5 | | 3.43 | 2.05 | 1 | 18 | 37.3% | 20.2% | 15.2% | 15.1% | 12.3% | 16.9% | 83.1% | 5333 |
| Nigeria | MICS4 | 0.80 | 1.05 | 0 | 7 | | 3.05 | 1.67 | 1 | 28 | 28.0% | 24.5% | 19.1% | 16.3% | 12.2% | 20.5% | 79.5% | 25044 |
| Palestine | MICS5 | 0.61 | 0.94 | 0 | 5 | | 2.91 | 1.87 | 1 | 16 | 23.1% | 19.5% | 21.4% | 19.8% | 16.2% | 72.9% | 16.1% | 7816 |
| Rwanda | DHS VII | 0.52 | 0.78 | 0 | 4 | | 2.36 | 1.02 | 1 | 14 | 24.2% | 21.0% | 19.1% | 17.1% | 18.6% | 21.4% | 78.6% | 7604 |
| Sao Tome and Principe | MICS5 | 0.58 | 0.78 | 0 | 4 | | 2.31 | 1.08 | 1 | 8 | 26.7% | 22.8% | 20.0% | 18.3% | 12.3% | 59.7% | 40.3% | 2016 |
| Serbia | MICS5 | 0.15 | 0.43 | 0 | 3 | | 3.05 | 1.35 | 1 | 11 | 14.5% | 16.9% | 20.0% | 21.5% | 27.2% | 62.8% | 37.2% | 2718 |
| Sierra Leone | MICS4 | 0.82 | 1.04 | 0 | 8 | | 3.77 | 2.18 | 1 | 19 | 22.9% | 20.8% | 19.9% | 19.8% | 16.5% | 29.5% | 70.5% | 8342 |
| Somalia | MICS4 | 0.82 | 1.12 | 0 | 6 | | 2.56 | 1.37 | 1 | 14 | 20.7% | 19.9% | 19.5% | 20.8% | 19.0% | 64.2% | 35.8% | 4674 |
| St Lucia | MICS4 | 0.39 | 0.62 | 0 | 4 | | 2.95 | 1.58 | 1 | 10 | 48.3% | 51.7% |  |  |  | 38.0% | 62.0% | 292 |
| Suriname | MICS4 | 0.60 | 0.85 | 0 | 6 | | 2.77 | 1.57 | 1 | 10 | 53.3% | 17.1% | 12.0% | 9.8% | 7.8% | 29.9% | 28.0% | 3287 |
| Swaziland | MICS5 | 0.88 | 1.08 | 0 | 8 | | 3.39 | 1.94 | 1 | 12 | 27.7% | 24.7% | 21.8% | 14.1% | 11.7% | 15.8% | 84.2% | 2675 |
| Thailand | MICS4 | 0.25 | 0.51 | 0 | 4 | | 3.17 | 1.42 | 1 | 13 | 14.6% | 18.6% | 22.3% | 23.6% | 20.9% | 51.6% | 48.4% | 9703 |
| Togo | DHS VI | 0.93 | 1.22 | 0 | 13 | | 3.06 | 1.74 | 1 | 18 | 30.6% | 20.7% | 18.9% | 15.2% | 14.7% | 27.9% | 72.1% | 6660 |
| Tunisia | MICS4 | 0.32 | 0.65 | 0 | 3 | | 2.52 | 1.18 | 1 | 13 | 26.5% | 22.1% | 17.5% | 20.4% | 13.5% | 57.8% | 42.2% | 2893 |
| Turkmenistan | MICS5 | 0.29 | 0.63 | 0 | 5 | | 4.28 | 1.99 | 1 | 12 | 17.3% | 18.9% | 21.2% | 23.6% | 19.0% | 41.7% | 58.3% | 3618 |
| Ukraine | MICS4 | 0.19 | 0.47 | 0 | 4 | | 2.69 | 1.10 | 1 | 8 | 21.9% | 22.4% | 16.9% | 18.9% | 20.0% | 63.2% | 36.8% | 4378 |
| Uruguay | MICS4 | 0.35 | 0.66 | 0 | 3 | | 2.45 | 1.06 | 1 | 8 | 31.6% | 17.2% | 15.4% | 14.6% | 21.1% | 88.5% | 11.5% | 1599 |
| Vietnam | MICS5 | 0.26 | 0.54 | 0 | 4 | | 3.47 | 1.59 | 1 | 11 | 25.0% | 17.7% | 18.6% | 19.8% | 18.9% | 38.1% | 61.9% | 3317 |
| Zimbabwe | MICS5 | 0.64 | 0.85 | 0 | 6 | | 2.59 | 1.32 | 1 | 18 | 20.9% | 21.2% | 18.3% | 21.8% | 17.7% | 28.4% | 71.6% | 9883 |

**Table S5.** Predictors of ‘Number of days child was home alone’ (Incidence Rate Ratio)

| **Country** | **Female** | | | **Age (in years)** | | | **Mother's level of education (lowest versus highest)** | | | **Number of children aged 10 - 14 years living in the household** | | | **Number of adults aged 15 years and older living in the household** | | | **Wealth score  (lowest versus highest)** | | | **Rural** | | | ***N*** |
| --- | --- | --- | --- | --- | --- | --- | --- | --- | --- | --- | --- | --- | --- | --- | --- | --- | --- | --- | --- | --- | --- | --- |
|  | IRR | LCI | UCI | IRR | LCI | UCI | IRR | LCI | UCI | IRR | LCI | UCI | IRR | LCI | UCI | IRR | LCI | UCI | IRR | CI | UCI |  |
| Afghanistan | 0.90 | 0.85 | 0.95 | 1.29 | 1.24 | 1.33 | 1.00 | 0.79 | 1.26 | 1.03 | 0.98 | 1.08 | 1.04 | 1.02 | 1.06 | 0.83 | 0.66 | 1.06 | 2.05 | 1.79 | 2.35 | 14442 |
| Algeria | 1.00 | 0.80 | 1.26 | 1.32 | 1.18 | 1.48 | 0.94 | 0.74 | 1.20 | 1.12 | 0.89 | 1.40 | 1.04 | 0.96 | 1.11 | 1.38 | 0.90 | 2.12 | 1.06 | 0.73 | 1.56 | 14593 |
| Bangladesh | 1.09 | 0.94 | 1.27 | 1.28 | 1.19 | 1.39 | 0.75 | 0.64 | 0.88 | 1.07 | 0.96 | 1.19 | 0.91 | 0.86 | 0.96 | 0.75 | 0.59 | 0.95 | 0.60 | 0.49 | 0.73 | 20712 |
| Barbados | 0.00 | 0.00 | 0.00 | 0.86 | 0.61 | 1.21 |  |  |  |  |  |  | 1.23 | 0.61 | 2.46 |  |  |  | 0.32 | 0.01 | 20.87 | 461 |
| Belarus | 1.50 | 0.44 | 5.11 | 1.87 | 1.00 | 3.49 | 1.75 | 0.44 | 6.99 | 0.10 | 0.01 | 1.26 | 0.43 | 0.23 | 0.81 | 0.38 | 0.05 | 2.83 | 6.05 | 2.35 | 15.55 | 3442 |
| Belize | 0.76 | 0.16 | 3.56 | 1.06 | 0.74 | 1.54 | 1.47 | 0.38 | 5.66 | 0.82 | 0.44 | 1.54 | 0.82 | 0.64 | 1.05 | 0.00 | 0.00 | 0.00 | 12.8 | 1.53 | 107.7 | 1941 |
| Benin | 0.96 | 0.88 | 1.04 | 1.33 | 1.28 | 1.38 | 0.93 | 0.78 | 1.10 | 1.07 | 1.00 | 1.16 | 1.00 | 0.97 | 1.03 | 0.67 | 0.55 | 0.82 | 1.31 | 1.18 | 1.46 | 12232 |
| Bhutan | 0.99 | 0.74 | 1.32 | 1.28 | 1.13 | 1.46 | 0.73 | 0.48 | 1.12 | 1.12 | 0.98 | 1.28 | 1.08 | 0.97 | 1.21 | 0.46 | 0.23 | 0.94 | 2.36 | 1.48 | 3.78 | 6241 |
| Bosnia and Herzegovina | 0.32 | 0.06 | 1.66 | 1.46 | 1.10 | 1.94 | 0.19 | 0.06 | 0.64 | 3.01 | 1.10 | 8.23 | 1.19 | 0.56 | 2.52 | 1.52 | 0.12 | 19.21 | 1.82 | 0.36 | 9.15 | 2296 |
| Cambodia | 0.82 | 0.59 | 1.14 | 1.52 | 1.30 | 1.77 | 0.32 | 0.21 | 0.51 | 1.17 | 0.96 | 1.43 | 0.63 | 0.56 | 0.71 | 0.29 | 0.10 | 0.82 | 2.56 | 1.65 | 3.97 | 7004 |
| Cameroon | 1.01 | 0.89 | 1.15 | 1.34 | 1.27 | 1.42 | 0.98 | 0.81 | 1.19 | 0.89 | 0.81 | 0.98 | 0.96 | 0.91 | 1.01 | 0.37 | 0.24 | 0.58 | 1.23 | 1.01 | 1.49 | 7015 |
| Central African Republic | 1.00 | 0.89 | 1.13 | 1.43 | 1.35 | 1.50 | 1.17 | 0.98 | 1.41 | 0.95 | 0.86 | 1.04 | 1.02 | 0.99 | 1.05 | 1.38 | 1.12 | 1.71 | 0.86 | 0.78 | 0.95 | 10220 |
| Chad | 1.00 | 0.92 | 1.08 | 1.09 | 1.07 | 1.11 | 0.78 | 0.66 | 0.93 | 1.03 | 0.98 | 1.09 | 1.00 | 0.96 | 1.03 | 0.84 | 0.61 | 1.16 | 1.02 | 0.87 | 1.18 | 10619 |
| Congo Democratic Republic | 0.92 | 0.79 | 1.07 | 1.21 | 1.16 | 1.26 | 0.95 | 0.77 | 1.17 | 1.13 | 1.03 | 1.25 | 1.03 | 0.96 | 1.10 | 0.48 | 0.32 | 0.72 | 1.20 | 0.99 | 1.45 | 8025 |
| Costa Rica | 0.93 | 0.29 | 2.93 | 0.79 | 0.57 | 1.10 | 1.97 | 0.63 | 6.15 | 0.83 | 0.42 | 1.64 | 0.96 | 0.67 | 1.40 | 2.59 | 0.43 | 15.45 | 0.63 | 0.23 | 1.75 | 2262 |
| Cuba | 1.29 | 0.71 | 2.34 | 1.07 | 0.89 | 1.27 | 0.53 | 0.13 | 2.11 | 0.80 | 0.43 | 1.48 | 0.63 | 0.45 | 0.87 |  |  |  | 2.16 | 0.75 | 6.23 | 5627 |
| Dominican Republic | 0.91 | 0.66 | 1.26 | 1.31 | 1.18 | 1.46 | 0.29 | 0.22 | 0.39 | 1.32 | 1.04 | 1.69 | 0.74 | 0.62 | 0.88 | 0.29 | 0.12 | 0.69 | 0.87 | 0.63 | 1.20 | 19835 |
| Egypt | 0.75 | 0.59 | 0.95 | 1.08 | 1.00 | 1.17 | 0.77 | 0.53 | 1.10 | 1.09 | 0.90 | 1.32 | 1.16 | 1.04 | 1.29 | 0.38 | 0.17 | 0.84 | 1.65 | 1.17 | 2.33 | 15843 |
| El Salvador | 0.78 | 0.50 | 1.23 | 1.21 | 1.06 | 1.39 | 1.02 | 0.56 | 1.83 | 0.99 | 0.73 | 1.34 | 1.16 | 1.02 | 1.32 | 1.08 | 0.47 | 2.44 | 0.78 | 0.51 | 1.21 | 7333 |
| Ghana | 0.97 | 0.81 | 1.16 | 1.31 | 1.20 | 1.43 | 0.71 | 0.55 | 0.92 | 1.05 | 0.91 | 1.21 | 1.06 | 1.00 | 1.12 | 0.80 | 0.52 | 1.25 | 1.20 | 0.91 | 1.57 | 7518 |
| Guinea-Bissau | 0.89 | 0.79 | 1.01 | 1.28 | 1.22 | 1.34 | 1.15 | 0.84 | 1.57 | 1.01 | 0.93 | 1.11 | 0.93 | 0.90 | 0.97 | 0.89 | 0.65 | 1.20 | 0.60 | 0.48 | 0.73 | 7445 |
| Guyana | 1.34 | 0.80 | 2.22 | 1.09 | 0.93 | 1.29 | 0.81 | 0.40 | 1.67 | 1.37 | 1.07 | 1.74 | 0.92 | 0.66 | 1.30 | 0.12 | 0.03 | 0.42 | 1.30 | 0.55 | 3.07 | 3355 |
| Honduras | 1.10 | 0.78 | 1.55 | 1.24 | 1.10 | 1.39 | 0.31 | 0.14 | 0.68 | 0.81 | 0.58 | 1.13 | 0.89 | 0.66 | 1.20 | 0.21 | 0.05 | 0.92 | 1.00 | 0.54 | 1.85 | 9973 |
| Iraq | 0.97 | 0.84 | 1.13 | 1.36 | 1.27 | 1.45 | 1.53 | 1.20 | 1.96 | 0.81 | 0.58 | 1.13 | 0.97 | 0.91 | 1.04 | 1.58 | 1.20 | 2.08 | 1.89 | 1.50 | 2.37 | 36309 |
| Jamaica | 2.00 | 0.54 | 7.47 | 0.72 | 0.35 | 1.45 | 0.12 | 0.02 | 0.71 | 1.12 | 0.67 | 1.86 | 1.12 | 0.95 | 1.32 | 0.00 | 0.00 | 0.00 | 0.30 | 0.06 | 1.45 | 1638 |
| Jordan | 1.29 | 0.58 | 2.88 | 1.17 | 1.00 | 1.36 | 2.57 | 0.96 | 6.87 | 0.68 | 0.55 | 0.85 | 1.19 | 0.81 | 1.77 | 0.42 | 0.15 | 1.16 | 1.33 | 0.69 | 2.57 | 10284 |
| Kazakhstan | 1.22 | 0.43 | 3.46 | 2.47 | 1.30 | 4.68 | 1.29 | 0.53 | 3.16 | 0.95 | 0.57 | 1.61 | 0.84 | 0.66 | 1.07 | 0.38 | 0.13 | 1.14 | 1.42 | 0.59 | 3.41 | 5504 |
| Kosovo under UNSC res. 1244 | 1.75 | 0.95 | 3.22 | 0.98 | 0.77 | 1.24 | 1.30 | 0.67 | 2.52 | 0.98 | 0.69 | 1.39 | 0.93 | 0.81 | 1.06 | 0.61 | 0.14 | 2.60 | 1.08 | 0.56 | 2.07 | 1646 |
| Kyrgyzstan | 1.04 | 0.50 | 2.17 | 1.66 | 1.06 | 2.61 | 1.09 | 0.46 | 2.60 | 1.55 | 0.76 | 3.18 | 0.64 | 0.34 | 1.20 | 0.73 | 0.14 | 3.97 | 0.89 | 0.35 | 2.28 | 4564 |
| Lao People's Democratic Republic | 0.85 | 0.69 | 1.06 | 1.64 | 1.45 | 1.84 | 0.95 | 0.56 | 1.63 | 0.85 | 0.74 | 0.99 | 0.97 | 0.88 | 1.06 | 1.42 | 0.83 | 2.41 | 3.08 | 1.98 | 4.81 | 10988 |
| Macedonia | 1.18 | 0.27 | 5.19 | 1.00 | 0.55 | 1.80 | 2.51 | 0.81 | 7.76 | 1.37 | 0.51 | 3.69 | 0.81 | 0.53 | 1.25 | 0.04 | 0.00 | 0.37 | 3.60 | 1.18 | 11.00 | 1367 |
| Madagascar | 0.90 | 0.76 | 1.07 | 1.41 | 1.21 | 1.65 | 2.02 | 1.46 | 2.81 | 1.16 | 1.03 | 1.30 | 1.06 | 0.96 | 1.17 | 3.36 | 1.82 | 6.22 | 1.41 | 0.78 | 2.52 | 2983 |
| Malawi | 0.92 | 0.84 | 1.01 | 1.57 | 1.50 | 1.65 | 0.85 | 0.76 | 0.96 | 1.03 | 0.97 | 1.09 | 0.93 | 0.89 | 0.97 | 0.54 | 0.42 | 0.70 | 1.18 | 0.95 | 1.47 | 18941 |
| Mali | 1.00 | 0.93 | 1.07 | 1.36 | 1.31 | 1.42 | 0.98 | 0.82 | 1.17 | 1.02 | 0.97 | 1.07 | 1.02 | 1.00 | 1.03 | 0.76 | 0.48 | 1.20 | 1.34 | 1.05 | 1.71 | 16100 |
| Mauritania | 0.85 | 0.74 | 0.99 | 1.25 | 1.18 | 1.33 | 0.52 | 0.41 | 0.66 | 1.02 | 0.91 | 1.15 | 1.01 | 0.97 | 1.06 | 0.92 | 0.64 | 1.33 | 1.17 | 0.92 | 1.49 | 8954 |
| Mexico | 0.89 | 0.61 | 1.30 | 1.11 | 0.91 | 1.35 | 0.24 | 0.14 | 0.41 | 1.35 | 1.00 | 1.84 | 0.98 | 0.84 | 1.13 | 0.89 | 0.36 | 2.18 | 1.09 | 0.55 | 2.13 | 8059 |
| Moldova | 2.12 | 0.81 | 5.56 | 2.65 | 1.68 | 4.19 | 1.81 | 0.56 | 5.80 | 0.15 | 0.04 | 0.60 | 0.76 | 0.36 | 1.63 | 0.63 | 0.13 | 3.11 | 1.71 | 0.57 | 5.11 | 1868 |
| Mongolia | 1.15 | 0.83 | 1.59 | 1.12 | 0.97 | 1.29 | 0.59 | 0.42 | 0.83 | 0.85 | 0.63 | 1.13 | 0.82 | 0.69 | 0.97 | 0.41 | 0.24 | 0.70 | 3.35 | 2.49 | 4.51 | 6051 |
| Montenegro | 3.06 | 1.30 | 7.23 | 1.35 | 1.00 | 1.83 | 3.05 | 0.22 | 42.40 | 1.03 | 0.83 | 1.29 | 0.81 | 0.38 | 1.75 | 0.11 | 0.01 | 0.87 | 0.00 | 0.00 | 0.00 | 1417 |
| Myanmar | 0.98 | 0.67 | 1.43 | 1.22 | 1.10 | 1.35 | 0.41 | 0.25 | 0.69 | 1.29 | 1.03 | 1.61 | 0.85 | 0.74 | 0.97 | 0.27 | 0.10 | 0.70 | 2.26 | 1.24 | 4.12 | 4666 |
| Nepal | 1.03 | 0.87 | 1.23 | 1.29 | 1.15 | 1.45 | 0.67 | 0.51 | 0.87 | 1.03 | 0.88 | 1.20 | 0.95 | 0.89 | 1.02 | 0.42 | 0.25 | 0.70 | 1.38 | 0.97 | 1.98 | 5333 |
| Nigeria | 1.00 | 0.92 | 1.09 | 1.49 | 1.43 | 1.57 | 0.95 | 0.83 | 1.08 | 1.01 | 0.96 | 1.06 | 1.01 | 0.98 | 1.04 | 1.11 | 0.94 | 1.31 | 1.04 | 0.91 | 1.19 | 25044 |
| Palestine | 1.17 | 0.91 | 1.49 | 1.14 | 1.01 | 1.29 | 1.31 | 0.91 | 1.88 | 1.04 | 0.86 | 1.26 | 0.93 | 0.83 | 1.04 | 1.24 | 0.59 | 2.58 | 1.41 | 0.95 | 2.09 | 7816 |
| Rwanda | 0.87 | 0.73 | 1.04 | 1.16 | 1.09 | 1.25 | 0.84 | 0.52 | 1.35 | 1.07 | 0.93 | 1.22 | 1.02 | 0.90 | 1.14 | 0.80 | 0.51 | 1.28 | 0.88 | 0.67 | 1.16 | 7604 |
| Sao Tome and Principe | 0.88 | 0.57 | 1.35 | 1.34 | 1.13 | 1.58 | 0.52 | 0.27 | 0.98 | 0.93 | 0.75 | 1.15 | 0.85 | 0.73 | 0.98 | 0.28 | 0.11 | 0.73 | 0.90 | 0.53 | 1.55 | 2016 |
| Serbia | 0.78 | 0.06 | 10.24 | 1.29 | 0.96 | 1.73 | 0.00 | 0.00 | 0.00 | 0.00 | 0.00 | 0.00 | 1.03 | 0.48 | 2.24 | 0.00 | 0.00 | 0.00 | 2.45 | 0.11 | 56.64 | 2718 |
| Sierra Leone | 0.95 | 0.85 | 1.05 | 1.36 | 1.30 | 1.41 | 1.15 | 0.94 | 1.40 | 0.94 | 0.87 | 1.02 | 1.02 | 0.98 | 1.06 | 1.65 | 1.27 | 2.14 | 1.32 | 1.10 | 1.60 | 8342 |
| Somalia | 0.93 | 0.75 | 1.15 | 1.36 | 1.25 | 1.48 | 0.79 | 0.49 | 1.30 | 0.95 | 0.86 | 1.05 | 0.95 | 0.88 | 1.02 | 0.40 | 0.26 | 0.62 | 0.95 | 0.77 | 1.18 | 4674 |
| St Lucia | 0.53 | 0.14 | 1.94 | 0.88 | 0.45 | 1.72 | 0.05 | 0.01 | 0.43 | 1.47 | 0.75 | 2.87 | 0.62 | 0.45 | 0.85 | 0.26 | 0.03 | 2.37 | 3.93 | 0.45 | 34.00 | 292 |
| Suriname | 1.40 | 0.63 | 3.10 | 1.36 | 0.72 | 2.56 | 1.19 | 0.80 | 1.78 | 0.97 | 0.68 | 1.40 | 1.28 | 0.91 | 1.80 | 1.81 | 0.34 | 9.80 | 0.86 | 0.30 | 2.42 | 3287 |
| Swaziland | 1.50 | 0.96 | 2.35 | 1.08 | 0.87 | 1.35 | 1.20 | 0.61 | 2.36 | 1.14 | 0.83 | 1.56 | 0.87 | 0.78 | 0.98 | 1.71 | 0.94 | 3.13 | 0.47 | 0.21 | 1.07 | 2675 |
| Thailand | 0.62 | 0.23 | 1.65 | 2.68 | 1.92 | 3.74 | 1.13 | 0.60 | 2.11 | 1.40 | 0.90 | 2.19 | 0.92 | 0.73 | 1.17 | 0.80 | 0.18 | 3.57 | 0.71 | 0.39 | 1.28 | 9703 |
| Togo | 1.18 | 1.05 | 1.33 | 1.13 | 1.07 | 1.19 | 0.82 | 0.61 | 1.10 | 1.13 | 1.05 | 1.21 | 1.01 | 0.96 | 1.06 | 0.93 | 0.61 | 1.42 | 0.74 | 0.63 | 0.86 | 6660 |
| Tunisia | 0.96 | 0.58 | 1.60 | 1.28 | 1.07 | 1.54 | 0.57 | 0.33 | 1.00 | 0.94 | 0.70 | 1.25 | 0.94 | 0.78 | 1.13 | 0.32 | 0.10 | 1.00 | 2.05 | 1.17 | 3.57 | 2893 |
| Turkmenistan | 5.32 | 2.09 | 13.56 | 1.35 | 0.71 | 2.55 | 0.83 | 0.14 | 4.85 | 0.90 | 0.27 | 3.01 | 1.60 | 1.23 | 2.08 | 1.34 | 0.02 | 101.1 | 0.98 | 0.17 | 5.71 | 3618 |
| Ukraine | 0.42 | 0.16 | 1.12 | 2.46 | 1.56 | 3.87 | 1.18 | 0.56 | 2.50 | 1.30 | 0.67 | 2.52 | 1.06 | 0.75 | 1.48 | 0.56 | 0.10 | 2.97 | 1.39 | 0.65 | 2.97 | 4378 |
| Uruguay | 1.21 | 0.46 | 3.18 | 1.45 | 1.13 | 1.87 | 0.51 | 0.12 | 2.14 | 0.41 | 0.22 | 0.78 | 0.68 | 0.33 | 1.40 | 2.39 | 0.66 | 8.71 | 0.58 | 0.24 | 1.36 | 1599 |
| Vietnam | 1.43 | 0.73 | 2.82 | 1.91 | 1.48 | 2.46 | 0.46 | 0.23 | 0.93 | 0.62 | 0.27 | 1.43 | 0.45 | 0.29 | 0.69 | 0.05 | 0.01 | 0.23 | 1.67 | 0.78 | 3.58 | 3317 |
| Zimbabwe | 0.81 | 0.59 | 1.09 | 1.71 | 1.56 | 1.89 | 1.14 | 0.84 | 1.56 | 1.10 | 0.93 | 1.31 | 0.76 | 0.68 | 0.84 | 0.45 | 0.22 | 0.93 | 1.65 | 1.22 | 2.22 | 9883 |

**Table S6.** Statistics per country of ‘Number of days child was supervised by another child,’ and descriptives of ‘Sex,’ ‘Age’ and ‘Mother’s level of education’ used in the models for ‘Number of days child was supervised by another child.’

| **Country** | **Survey** | **Number of days child was supervised by another child** | | | | | | | | **Sex** | | **Age in years** | | | | **Mother's level of education (percentage)** | | ***N*** |
| --- | --- | --- | --- | --- | --- | --- | --- | --- | --- | --- | --- | --- | --- | --- | --- | --- | --- | --- |
|  |  | **0** | **1** | **2** | **3** | **4** | **5** | **6** | **7** | **Male** | **Female** | **Mean** | **SD** | **Min** | **Max** | **1** | **2** |  |
| Afghanistan | MICS4 | 71.0% | 5.3% | 8.8% | 5.4% | 3.1% | 1.7% | 2.3% | 2.5% | 51.1% | 48.9% | 3.11 | 1.39 | 1 | 5 | 94.2% | 5.8% | 14127 |
| Algeria | MICS4 | 96.9% | 1.1% | 1.1% | 0.4% | 0.2% | 0.1% | 0.1% | 0.1% | 51.8% | 48.2% | 2.86 | 1.41 | 1 | 5 | 69.2% | 30.8% | 14562 |
| Bangladesh | MICS5 | 92.0% | 1.7% | 1.9% | 1.2% | 0.9% | 0.6% | 0.7% | 1.1% | 51.3% | 48.7% | 2.99 | 1.42 | 1 | 5 | 39.7% | 60.3% | 20692 |
| Barbados | MICS4 | 99.8% |  |  | 0.2% |  |  |  |  | 55.1% | 44.9% | 3.13 | 1.43 | 1 | 5 |  |  | 461 |
| Belarus | MICS4 | 96.7% | 1.8% | 0.8% | 0.3% | 0.1% | 0.1% | 0.1% | 0.1% | 51.4% | 48.6% | 2.98 | 1.41 | 1 | 5 | 18.8% | 81.2% | 3441 |
| Belize | MICS4 | 98.2% | 0.8% | 0.2% | 0.4% | 0.3% | 0.1% | 0.1% | 0.1% | 51.2% | 48.8% | 2.98 | 1.40 | 1 | 5 | 56.6% | 43.4% | 1939 |
| Benin | MICS5 | 79.1% | 5.1% | 8.1% | 2.3% | 1.3% | 1.3% | 0.8% | 1.9% | 49.3% | 50.7% | 2.93 | 1.42 | 1 | 5 | 83.8% | 16.2% | 12234 |
| Bhutan | MICS4 | 90.1% | 3.1% | 2.7% | 1.4% | 0.5% | 1.0% | 0.6% | 0.6% | 51.4% | 48.6% | 2.90 | 1.38 | 1 | 5 | 80.6% | 19.4% | 6236 |
| Bosnia and Herzegovina | MICS4 | 98.6% | 0.5% | 0.1% | 0.3% | 0.0% | 0.2% | 0.41 | 0.20 | 0.83 | 50.9% | 3.18 | 1.31 | 1 | 5 | 22.7% | 77.3% | 2293 |
| Cambodia | DHS VII | 92.9% | 1.4% | 1.5% | 1.0% | 0.8% | 0.8% | 0.4% | 1.2% | 50.3% | 49.7% | 3.62 | 1.53 | 1 | 5 | 63.8% | 36.2% | 6991 |
| Cameroon | MICS5 | 73.3% | 4.4% | 6.3% | 4.6% | 2.6% | 2.5% | 2.3% | 4.1% | 50.9% | 49.1% | 2.91 | 1.40 | 1 | 5 | 63.3% | 36.7% | 7004 |
| Central African Republic | MICS4 | 49.4% | 4.4% | 9.2% | 9.2% | 6.1% | 4.9% | 9.9% | 6.9% | 49.2% | 50.8% | 2.77 | 1.40 | 1 | 5 | 88.8% | 11.2% | 10025 |
| Chad | DHS VII | 51.7% | 5.9% | 10.5% | 9.7% | 7.6% | 5.9% | 4.3% | 4.4% | 50.3% | 49.7% | 3.10 | 1.49 | 1 | 5 | 91.9% | 8.1% | 10405 |
| Congo Democratic Republic | DHS VI | 54.1% | 4.7% | 7.7% | 8.9% | 6.0% | 5.8% | 8.2% | 4.6% | 49.4% | 50.6% | 1.90 | 1.42 | 0 | 4 | 65.7% | 34.3% | 7890 |
| Costa Rica | MICS4 | 97.7% | 0.8% | 0.1% | 0.1% | 0.9% | 0.3% |  | 0.1% | 50.4% | 49.6% | 2.91 | 1.40 | 1 | 5 | 39.4% | 60.6% | 2259 |
| Cuba | MICS5 | 99.4% | 0.2% | 0.2% | 0.0% | 0.1% | 0.0% |  | 0.1% | 50.4% | 49.6% | 2.97 | 1.37 | 1 | 5 | 20.4% | 79.6% | 5626 |
| Dominican Republic | MICS5 | 97.5% | 1.0% | 0.6% | 0.3% | 0.1% | 0.2% | 0.2% | 0.1% | 50.8% | 49.2% | 2.95 | 1.41 | 1 | 5 | 36.4% | 63.6% | 19648 |
| Egypt | DHS VI | 98.0% | 0.5% | 0.5% | 0.4% | 0.2% | 0.1% | 0.1% | 0.2% | 52.0% | 48.0% | 1.89 | 1.40 | 0 | 4 | 25.1% | 74.9% | 15843 |
| El Salvador | MICS5 | 98.6% | 0.8% | 0.4% | 0.1% | 0.0% | 0.1% | 0.0% | 0.0% | 50.7% | 49.3% | 3.02 | 1.41 | 1 | 5 | 39.8% | 60.2% | 7333 |
| Ghana | MICS4 | 85.8% | 1.9% | 2.8% | 3.2% | 1.9% | 1.4% | 0.9% | 2.2% | 51.1% | 48.9% | 2.95 | 1.41 | 1 | 5 | 76.2% | 23.8% | 7531 |
| Guinea-Bissau | MICS5 | 80.4% | 2.3% | 2.8% | 2.5% | 2.6% | 3.4% | 2.3% | 3.8% | 50.6% | 49.4% | 2.93 | 1.42 | 1 | 5 | 88.4% | 11.6% | 7395 |
| Guyana | MICS5 | 96.6% | 1.3% | 1.0% | 0.3% | 0.3% | 0.1% | 0.2% | 0.2% | 50.5% | 49.5% | 2.95 | 1.40 | 1 | 5 | 19.9% | 80.1% | 3351 |
| Honduras | DHS VI | 95.8% | 2.3% | 0.9% | 0.5% | 0.1% | 0.1% | 0.1% | 0.2% | 52.0% | 48.0% | 2.91 | 1.44 | 1 | 5 | 69.2% | 30.8% | 9961 |
| Iraq | MICS4 | 93.7% | 1.5% | 1.7% | 1.0% | 0.5% | 0.5% | 0.2% | 0.7% | 51.1% | 48.9% | 2.88 | 1.40 | 1 | 5 | 74.8% | 25.2% | 36299 |
| Jamaica | MICS4 | 99.2% | 0.2% | 0.2% | 0.1% |  | 0.1% | 0.2% |  | 52.5% | 47.5% | 2.99 | 1.46 | 1 | 5 | 79.2% | 20.8% | 1632 |
| Jordan | DHS VI | 93.0% | 4.0% | 1.5% | 0.7% | 0.2% | 0.3% | 0.1% | 0.2% | 52.1% | 47.9% | 3.70 | 1.48 | 1 | 5 | 10.0% | 90.0% | 10284 |
| Kazakhstan | MICS5 | 96.4% | 1.7% | 0.9% | 0.4% | 0.1% | 0.2% | 0.0% | 0.1% | 51.4% | 48.6% | 2.97 | 1.42 | 1 | 5 | 26.7% | 73.3% | 5503 |
| Kosovo under UNSC res. 1244 | MICS5 | 96.4% | 2.6% | 0.6% | 0.3% | 0.1% | 0.1% |  | 0.1% | 53.0% | 47.0% | 3.00 | 1.41 | 1 | 5 | 53.8% | 46.2% | 1646 |
| Kyrgyzstan | MICS5 | 96.4% | 0.9% | 1.4% | 0.5% | 0.6% | 0.0% | 0.0% | 0.1% | 51.2% | 48.8% | 2.93 | 1.43 | 1 | 5 | 57.2% | 42.8% | 4559 |
| Lao People's Democratic Republic | MICS4 | 89.2% | 1.9% | 3.5% | 2.1% | 1.4% | 1.0% | 0.3% | 0.7% | 50.8% | 49.2% | 2.90 | 1.42 | 1 | 5 | 75.7% | 24.3% | 10807 |
| Macedonia | MICS4 | 96.8% | 1.2% | 0.9% | 0.5% | 0.2% | 0.1% | 0.1% | 0.2% | 51.3% | 48.7% | 3.02 | 1.40 | 1 | 5 | 77.5% | 22.5% | 1359 |
| Madagascar | MICS4 | 75.6% | 2.8% | 5.0% | 4.1% | 3.0% | 1.9% | 1.5% | 6.2% | 50.3% | 49.7% | 2.84 | 1.41 | 1 | 5 | 89.9% | 10.1% | 2982 |
| Malawi | MICS5 | 66.2% | 7.8% | 8.7% | 6.7% | 3.4% | 2.8% | 1.6% | 3.0% | 50.0% | 50.0% | 3.00 | 1.39 | 1 | 5 | 83.5% | 16.5% | 18905 |
| Mali | MICS5 | 77.5% | 4.8% | 7.5% | 2.5% | 1.7% | 1.5% | 0.7% | 3.7% | 51.3% | 48.7% | 2.92 | 1.39 | 1 | 5 | 88.6% | 11.4% | 16061 |
| Mauritania | MICS4 | 82.1% | 3.2% | 5.4% | 2.6% | 1.5% | 1.8% | 1.7% | 1.7% | 50.9% | 49.1% | 2.91 | 1.42 | 1 | 5 | 60.1% | 39.9% | 8852 |
| Mexico | MICS5 | 96.3% | 2.1% | 0.8% | 0.3% | 0.1% | 0.2% | 0.1% | 0.1% | 50.5% | 49.5% | 3.01 | 1.42 | 1 | 5 | 23.0% | 77.0% | 8054 |
| Moldova | MICS4 | 95.0% | 2.4% | 1.0% | 0.9% | 0.2% | 0.4% | 0.1% | 0.1% | 51.8% | 48.2% | 2.89 | 1.41 | 1 | 5 | 41.6% | 58.4% | 1864 |
| Mongolia | MICS5 | 91.7% | 4.1% | 2.4% | 0.4% | 0.2% | 0.4% |  | 0.6% | 51.2% | 48.8% | 2.90 | 1.41 | 1 | 5 | 13.2% | 86.8% | 6045 |
| Montenegro | MICS5 | 98.0% | 0.9% | 0.6% | 0.3% |  | 0.2% |  | 0.1% | 52.5% | 47.5% | 3.02 | 1.39 | 1 | 5 | 16.0% | 84.0% | 1417 |
| Myanmar | DHS VII | 88.6% | 3.4% | 2.1% | 1.9% | 0.6% | 1.2% | 0.3% | 1.9% | 52.3% | 47.7% | 2.96 | 1.43 | 1 | 5 | 62.1% | 37.9% | 4664 |
| Nepal | MICS5 | 83.2% | 2.3% | 3.4% | 2.7% | 1.7% | 1.5% | 0.5% | 4.6% | 52.4% | 47.6% | 3.01 | 1.41 | 1 | 5 | 61.7% | 38.3% | 5307 |
| Nigeria | MICS4 | 64.6% | 6.1% | 10.2% | 6.9% | 4.2% | 3.9% | 2.2% | 1.9% | 51.1% | 48.9% | 2.90 | 1.43 | 1 | 5 | 68.9% | 31.1% | 24855 |
| Palestine | MICS5 | 88.5% | 5.4% | 2.8% | 1.7% | 0.5% | 0.6% | 0.2% | 0.3% | 52.1% | 47.9% | 3.02 | 1.40 | 1 | 5 | 30.4% | 69.6% | 7813 |
| Panama | MICS5 | 97.6% | 1.0% | 0.7% | 0.4% | 0.1% | 0.1% | 0.0% |  | 52.4% | 47.6% | 2.94 | 1.40 | 1 | 5 | 44.6% | 55.4% | 5794 |
| Rwanda | DHS VII | 69.4% | 3.9% | 6.4% | 6.1% | 3.1% | 3.4% | 3.7% | 4.0% | 50.6% | 49.4% | 3.97 | 1.44 | 1 | 5 | 86.3% | 13.7% | 7580 |
| Sao Tome and Principe | MICS5 | 86.4% | 3.8% | 3.5% | 1.4% | 1.1% | 1.6% | 0.8% | 1.5% | 50.4% | 49.6% | 3.07 | 1.40 | 1 | 5 | 70.0% | 30.0% | 1991 |
| Serbia | MICS5 | 99.2% | 0.3% | 0.4% | 0.0% |  | 0.0% |  | 0.0% | 50.3% | 49.7% | 3.13 | 1.41 | 1 | 5 | 63.4% | 36.6% | 2718 |
| Sierra Leone | MICS4 | 81.5% | 3.4% | 5.2% | 3.6% | 2.7% | 1.2% | 0.8% | 1.5% | 49.6% | 50.4% | 2.95 | 1.43 | 1 | 5 | 85.2% | 14.8% | 8290 |
| Somalia | MICS4 | 75.7% | 3.6% | 5.6% | 6.1% | 3.6% | 2.6% | 0.8% | 2.1% | 50.5% | 49.5% | 3.00 | 1.40 | 1 | 5 | 93.7% | 6.3% | 4607 |
| St Lucia | MICS4 | 97.3% | 2.7% |  |  |  |  |  |  | 49.7% | 50.3% | 2.96 | 1.40 | 1 | 5 |  |  | 292 |
| Suriname | MICS4 | 96.1% | 2.4% | 0.9% | 0.2% | 0.1% | 0.2% | 0.1% | 0.1% | 49.6% | 50.4% | 2.89 | 1.39 | 1 | 5 | 57.2% | 42.8% | 3224 |
| Swaziland | MICS5 | 89.2% | 3.6% | 2.3% | 1.7% | 1.2% | 1.5% | 0.2% | 0.3% | 51.1% | 48.9% | 2.94 | 1.38 | 1 | 5 | 41.7% | 58.3% | 2679 |
| Thailand | MICS4 | 96.2% | 1.7% | 1.1% | 0.3% | 0.1% | 0.2% | 0.0% | 0.4% | 51.0% | 49.0% | 3.15 | 1.36 | 1 | 5 | 38.6% | 61.4% | 9687 |
| Togo | DHS VI | 76.6% | 3.4% | 6.6% | 5.6% | 3.4% | 1.9% | 1.0% | 1.5% | 50.3% | 49.7% | 3.96 | 1.45 | 1 | 5 | 79.9% | 20.1% | 6611 |
| Tunisia | MICS4 | 89.3% | 4.1% | 3.1% | 1.5% | 0.8% | 0.4% | 0.4% | 0.5% | 53.3% | 46.7% | 2.92 | 1.40 | 1 | 5 | 50.2% | 49.8% | 2889 |
| Turkmenistan | MICS5 | 99.6% | 0.2% | 0.1% |  |  |  |  | 0.1% | 52.6% | 47.4% | 2.97 | 1.40 | 1 | 5 | 88.7% | 11.3% | 3757 |
| Ukraine | MICS4 | 94.0% | 1.9% | 1.7% | 1.2% | 0.4% | 0.3% | 0.1% | 0.5% | 50.9% | 49.1% | 3.10 | 1.37 | 1 | 5 | 35.7% | 64.3% | 4376 |
| Uruguay | MICS4 | 97.6% | 0.9% | 1.1% | 0.1% | 0.1% | 0.1% | 0.1% | 0.1% | 52.3% | 47.8% | 3.21 | 1.33 | 1 | 5 | 21.2% | 78.8% | 1600 |
| Vietnam | MICS5 | 94.0% | 1.7% | 2.3% | 0.7% | 0.3% | 0.2% | 0.3% | 0.6% | 51.6% | 48.4% | 2.80 | 1.41 | 1 | 5 | 23.1% | 76.9% | 3318 |
| Zimbabwe | MICS5 | 83.9% | 6.4% | 4.0% | 2.2% | 1.0% | 0.9% | 0.3% | 1.3% | 49.9% | 50.1% | 2.96 | 1.37 | 1 | 5 | 38.9% | 61.1% | 9880 |

**Table S7.** Descriptives of ‘Number of children aged 10 - 14 years living in the household,’ ‘Number of girls aged 10 - 14 years living in the household,’ ‘Number of adults aged 15 years and older living in the household,’ ‘Wealth index (percentage)’ and ‘Residence’ used in the models for ‘Number of days child was supervised by another child.’

| **Country** | **Number of children aged 10 - 14 years living in the household** | | | | **Number of girls aged 10 - 14 years living in the household** | | | | **Number of adults aged 15 years and older living in the household** | | | | **Wealth index (percentage)** | | | | | **Residence** | | ***N*** |
| --- | --- | --- | --- | --- | --- | --- | --- | --- | --- | --- | --- | --- | --- | --- | --- | --- | --- | --- | --- | --- |
|  | **Mean** | **SD** | **Min** | **Max** | **Mean** | **SD** | **Min** | **Max** | **Mean** | **SD** | **Min** | **Max** | **1** | **2** | **3** | **4** | **5** | **Urban** | **Rural** |  |
| Afghanistan | 1.06 | 1.13 | 0 | 6 | 0.52 | 0.787 | 0 | 6 | 4.42 | 2.60 | 1 | 23 | 19.0% | 20.0% | 19.1% | 19.6% | 22.2% | 24.3% | 75.7% | 14127 |
| Algeria | 0.47 | 0.74 | 0 | 4 | 0.23 | 0.5 | 0 | 3 | 3.49 | 2.17 | 1 | 16 | 23.5% | 20.5% | 20.7% | 19.5% | 15.8% | 64.6% | 35.4% | 14562 |
| Bangladesh | 0.46 | 0.70 | 0 | 6 | 0.24 | 0.508 | 0 | 4 | 3.14 | 1.68 | 1 | 16 | 30.1% | 21.7% | 18.1% | 16.2% | 14.0% | 15.9% | 84.1% | 20692 |
| Barbados |  |  |  |  |  |  |  |  |  |  |  |  |  |  |  |  |  | 59.2% | 40.8% | 461 |
| Belarus | 0.18 | 0.43 | 0 | 3 | 0.09 | 0.313 | 0 | 3 | 2.33 | 0.82 | 1 | 8 | 14.6% | 19.2% | 17.9% | 21.4% | 26.9% | 72.0% | 28.0% | 3441 |
| Belize | 0.61 | 0.89 | 0 | 4 | 0.30 | 0.597 | 0 | 4 | 2.93 | 1.54 | 1 | 11 | 29.6% | 22.6% | 20.0% | 16.0% | 11.8% | 35.2% | 64.8% | 1939 |
| Benin | 0.88 | 1.16 | 0 | 15 | 0.42 | 1 | 0 | 5 | 3.20 | 2.17 | 1 | 29 | 19.1% | 18.4% | 18.6% | 19.4% | 24.5% | 50.2% | 49.8% | 12234 |
| Bhutan | 0.50 | 0.74 | 0 | 4 | 0.26 | 0.53 | 0 | 4 | 3.53 | 1.69 | 1 | 14 | 21.7% | 21.6% | 20.5% | 20.8% | 15.4% | 21.8% | 78.2% | 6236 |
| Bosnia and Herzegovina | 0.22 | 0.50 | 0 | 4 | 0.13 | 0 | 0 | 2 | 2.91 | 1.21 | 1 | 8 | 17.2% | 20.2% | 21.1% | 18.6% | 22.9% | 34.9% | 65.1% | 2293 |
| Cambodia | 0.41 | 0.69 | 0 | 4 | 0.20 | 0 | 0 | 3 | 3.30 | 1.74 | 1 | 14 | 23.9% | 18.8% | 15.9% | 17.5% | 24.0% | 26.9% | 73.1% | 6991 |
| Cameroon | 0.84 | 1.07 | 0 | 8 | 0.42 | 0.692 | 0 | 6 | 3.21 | 1.81 | 1 | 16 | 17.2% | 23.2% | 23.0% | 20.2% | 16.4% | 45.9% | 54.1% | 7004 |
| Central African Republic | 0.67 | 0.93 | 0 | 10 | 0.34 | 0.631 | 0 | 6 | 2.75 | 1.46 | 1 | 12 | 22.3% | 25.0% | 24.1% | 18.0% | 10.6% | 31.9% | 68.1% | 10025 |
| Chad | 0.92 | 1.14 | 0 | 8 | 0.46 | 0.738 | 0 | 5 | 2.70 | 1.51 | 1 | 17 | 20.0% | 20.3% | 21.1% | 22.3% | 16.3% | 19.7% | 80.3% | 10405 |
| Congo Democratic Republic | 0.81 | 0.96 | 0 | 6 | 0.40 | 0.641 | 0 | 4 | 2.71 | 1.37 | 0 | 15 | 25.8% | 22.2% | 21.3% | 17.3% | 13.5% | 29.7% | 70.3% | 7890 |
| Costa Rica | 0.40 | 0.66 | 0 | 4 | 0.22 | 0.471 | 0 | 3 | 2.81 | 1.40 | 1 | 16 | 35.4% | 24.4% | 16.5% | 13.9% | 9.9% | 48.2% | 51.8% | 2259 |
| Cuba | 0.18 | 0.42 | 0 | 3 | 0.09 | 0.303 | 0 | 2 | 2.94 | 1.28 | 1 | 11 |  |  |  |  |  | 75.9% | 24.1% | 5626 |
| Dominican Republic | 0.34 | 0.64 | 0 | 5 | 0.17 | 0.436 | 0 | 3 | 2.55 | 1.21 | 1 | 10 | 31.0% | 22.8% | 18.5% | 15.1% | 12.6% | 64.3% | 35.7% | 19648 |
| Egypt | 0.37 | 0.71 | 0 | 5 | 0.19 | 0.475 | 0 | 4 | 2.64 | 1.49 | 1 | 16 | 18.4% | 18.7% | 20.6% | 20.8% | 21.5% | 40.9% | 59.1% | 15843 |
| El Salvador | 0.42 | 0.70 | 0 | 6 | 0.21 | 0.483 | 0 | 4 | 2.92 | 1.43 | 1 | 11 | 23.7% | 21.1% | 20.2% | 18.3% | 16.7% | 56.2% | 43.8% | 7333 |
| Ghana | 0.91 | 1.06 | 0 | 8 | 0.44 | 0.704 | 0 | 6 | 2.99 | 1.60 | 1 | 14 | 46.6% | 20.0% | 13.9% | 11.1% | 8.5% | 28.1% | 71.9% | 7531 |
| Guinea-Bissau | 1.13 | 1.22 | 0 | 9 | 0.54 | 0.76 | 0 | 5 | 5.06 | 3.02 | 1 | 24 | 30.4% | 24.5% | 22.8% | 14.2% | 8.0% | 25.8% | 74.2% | 7395 |
| Guyana | 0.53 | 0.80 | 0 | 4 | 0.27 | 0.538 | 0 | 3 | 3.05 | 1.64 | 1 | 13 | 37.9% | 19.1% | 15.6% | 13.5% | 13.9% | 20.6% | 79.4% | 3351 |
| Honduras | 0.68 | 0.90 | 0 | 7 | 0.33 | 0.6 | 0 | 4 | 3.09 | 1.61 | 0 | 13 | 33.0% | 23.2% | 17.8% | 15.3% | 10.7% | 33.7% | 66.3% | 9961 |
| Iraq | 0.83 | 1.14 | 0 | 8 | 0.42 | 0.744 | 0 | 6 | 4.13 | 2.79 | 1 | 23 | 34.2% | 23.9% | 18.4% | 13.6% | 9.8% | 54.1% | 45.9% | 36299 |
| Jamaica | 0.54 | 0.78 | 0 | 5 | 0.26 | 0.526 | 0 | 4 | 3.07 | 1.67 | 1 | 12 | 23.5% | 23.0% | 21.5% | 17.6% | 14.4% | 59.4% | 40.6% | 1632 |
| Jordan | 0.58 | 0.91 | 0 | 6 | 0.30 | 0.611 | 0 | 4 | 2.68 | 1.43 | 1 | 14 | 27.0% | 26.7% | 22.6% | 16.3% | 7.5% | 69.0% | 31.0% | 10284 |
| Kazakhstan | 0.31 | 0.59 | 0 | 3 | 0.16 | 0.422 | 0 | 3 | 3.17 | 1.43 | 1 | 12 | 19.6% | 18.9% | 22.3% | 19.8% | 19.5% | 55.2% | 44.8% | 5503 |
| Kosovo under UNSC res. 1244 | 0.50 | 0.92 | 0 | 6 | 0.27 | 0.635 | 0 | 4 | 4.74 | 2.45 | 1 | 20 | 24.2% | 19.2% | 19.7% | 19.3% | 17.7% | 35.8% | 64.2% | 1646 |
| Kyrgyzstan | 0.37 | 0.65 | 0 | 4 | 0.18 | 0.441 | 0 | 3 | 3.30 | 1.41 | 1 | 9 | 24.3% | 22.0% | 21.2% | 18.2% | 14.3% | 32.9% | 67.1% | 4559 |
| Lao People's Democratic Republic | 0.67 | 0.90 | 0 | 7 | 0.34 | 0.613 | 0 | 5 | 3.58 | 1.83 | 1 | 15 | 32.7% | 22.3% | 18.1% | 14.9% | 11.9% | 19.0% | 81.0% | 10807 |
| Macedonia | 0.16 | 0.47 | 0 | 3 | 0.09 | 0.328 | 0 | 3 | 3.58 | 1.68 | 1 | 17 | 18.0% | 19.9% | 21.4% | 19.6% | 21.2% | 54.7% | 45.3% | 1359 |
| Madagascar | 0.82 | 1.02 | 0 | 7 | 0.40 | 0.679 | 0 | 5 | 2.72 | 1.56 | 1 | 17 | 25.6% | 20.3% | 22.3% | 20.4% | 11.5% | 4.6% | 95.4% | 2982 |
| Malawi | 0.70 | 0.86 | 0 | 8 | 0.36 | 0.601 | 0 | 4 | 2.43 | 1.07 | 1 | 10 | 22.6% | 22.0% | 21.5% | 18.7% | 15.2% | 11.2% | 88.8% | 18905 |
| Mali | 1.68 | 1.82 | 0 | 14 | 0.82 | 1.081 | 0 | 8 | 5.92 | 4.60 | 1 | 35 | 21.4% | 20.5% | 19.6% | 20.2% | 18.3% | 21.4% | 78.6% | 16061 |
| Mauritania | 0.87 | 1.02 | 0 | 7 | 0.45 | 0.699 | 0 | 5 | 3.45 | 2.23 | 1 | 26 | 24.0% | 23.2% | 18.9% | 17.4% | 16.4% | 35.0% | 65.0% | 8852 |
| Mexico | 0.36 | 0.65 | 0 | 4 | 0.18 | 0.451 | 0 | 4 | 2.82 | 1.38 | 1 | 11 | 29.4% | 25.9% | 20.0% | 15.7% | 9.0% | 64.1% | 35.9% | 8054 |
| Moldova | 0.20 | 0.47 | 0 | 3 | 0.10 | 0.319 | 0 | 2 | 2.36 | 0.94 | 1 | 7 | 13.3% | 16.9% | 16.7% | 18.9% | 34.2% | 55.0% | 45.0% | 1864 |
| Mongolia | 0.32 | 0.58 | 0 | 4 | 0.16 | 0.406 | 0 | 3 | 2.52 | 1.01 | 1 | 13 | 24.0% | 20.7% | 19.2% | 17.3% | 18.9% | 58.1% | 41.9% | 6045 |
| Montenegro | 0.23 | 0.56 | 0 | 3 | 0.13 | 0.401 | 0 | 3 | 2.87 | 1.31 | 1 | 9 | 18.9% | 20.3% | 18.7% | 20.5% | 21.6% | 63.3% | 36.7% | 1417 |
| Myanmar | 0.53 | 0.77 | 0 | 5 | 0.27 | 0.534 | 0 | 3 | 3.24 | 1.72 | 1 | 17 | 30.2% | 22.8% | 17.9% | 16.7% | 12.3% | 21.1% | 78.9% | 4664 |
| Nepal | 0.50 | 0.79 | 0 | 5 | 0.31 | 0.608 | 0 | 4 | 3.43 | 2.04 | 1 | 18 | 37.3% | 20.2% | 15.2% | 15.1% | 12.3% | 16.9% | 83.1% | 5307 |
| Nigeria | 0.80 | 1.05 | 0 | 7 | 0.41 | 0.69 | 0 | 5 | 3.05 | 1.67 | 1 | 28 | 28.1% | 24.4% | 19.1% | 16.2% | 12.2% | 20.5% | 79.5% | 24855 |
| Palestine | 0.61 | 0.94 | 0 | 5 | 0.32 | 0.629 | 0 | 4 | 2.91 | 1.87 | 1 | 16 | 23.1% | 19.5% | 21.4% | 19.8% | 16.2% | 72.9% | 16.1% | 7813 |
| Panama | 0.71 | 0.92 | 0 | 6 | 0.34 | 0.602 | 0 | 3 | 3.25 | 1.73 | 1 | 14 | 52.1% | 18.2% | 13.0% | 10.4% | 6.4% | 33.3% | 66.7% | 5794 |
| Rwanda | 0.52 | 0.78 | 0 | 4 | 0.27 | 0.538 | 0 | 3 | 2.36 | 1.02 | 1 | 14 | 24.2% | 21.0% | 19.1% | 17.1% | 18.6% | 21.5% | 78.5% | 7580 |
| Sao Tome and Principe | 0.58 | 0.77 | 0 | 4 | 0.31 | 0.555 | 0 | 3 | 2.32 | 1.08 | 1 | 8 | 26.2% | 22.9% | 20.0% | 18.5% | 12.3% | 59.7% | 40.3% | 1991 |
| Serbia | 0.15 | 0.43 | 0 | 3 | 0.08 | 0.295 | 0 | 3 | 3.05 | 1.35 | 1 | 11 | 14.5% | 16.9% | 20.0% | 21.5% | 27.2% | 62.8% | 37.2% | 2718 |
| Sierra Leone | 0.82 | 1.04 | 0 | 8 | 0.44 | 0.729 | 0 | 8 | 3.76 | 2.17 | 1 | 19 | 23.1% | 20.8% | 19.9% | 19.7% | 16.6% | 29.5% | 70.5% | 8290 |
| Somalia | 0.82 | 1.12 | 0 | 6 | 0.41 | 0.702 | 0 | 5 | 2.56 | 1.37 | 1 | 14 | 20.5% | 20.0% | 19.4% | 21.0% | 19.1% | 64.2% | 35.8% | 4607 |
| St Lucia |  |  |  |  |  |  |  |  | 2.95 | 1.58 | 1 | 10 |  |  |  |  |  |  |  | 292 |
| Suriname | 0.60 | 0.86 | 0 | 6 | 0.28 | 0.562 | 0 | 4 | 2.77 | 1.57 | 1 | 10 | 53.8% | 16.7% | 11.9% | 9.9% | 7.8% | 29.6% | 28.1% | 3224 |
| Swaziland | 0.88 | 1.08 | 0 | 8 | 0.43 | 0.698 | 0 | 4 | 3.39 | 1.94 | 1 | 12 | 27.7% | 24.6% | 21.7% | 14.2% | 11.8% | 15.9% | 84.1% | 2679 |
| Thailand | 0.25 | 0.51 | 0 | 4 | 0.13 | 0.368 | 0 | 3 | 3.17 | 1.42 | 1 | 13 | 14.6% | 18.6% | 22.3% | 23.6% | 20.9% | 51.5% | 48.5% | 9687 |
| Togo | 0.93 | 1.22 | 0 | 13 | 0.47 | 0.793 | 0 | 7 | 3.07 | 1.74 | 1 | 18 | 30.4% | 20.7% | 18.9% | 15.2% | 14.8% | 28.1% | 71.9% | 6611 |
| Tunisia | 0.32 | 0.65 | 0 | 3 | 0.16 | 0.436 | 0 | 3 | 2.52 | 1.18 | 1 | 13 | 26.7% | 22.1% | 17.4% | 20.3% | 13.5% | 57.8% | 42.2% | 2889 |
| Turkmenistan | 0.29 | 0.63 | 0 | 5 | 0.15 | 0.438 | 0 | 3 | 4.24 | 2.00 | 1 | 12 | 16.7% | 18.2% | 20.8% | 23.4% | 20.9% | 43.4% | 56.6% | 3757 |
| Ukraine | 0.19 | 0.47 | 0 | 4 | 0.09 | 0.325 | 0 | 3 | 2.69 | 1.10 | 1 | 8 | 21.9% | 22.3% | 16.9% | 18.9% | 19.9% | 63.3% | 36.7% | 4376 |
| Uruguay | 0.35 | 0.66 | 0 | 3 | 0.18 | 0.45 | 0 | 3 | 2.45 | 1.06 | 1 | 8 | 31.6% | 17.2% | 15.4% | 14.6% | 21.1% | 88.5% | 11.5% | 1600 |
| Vietnam | 0.26 | 0.54 | 0 | 4 | 0.13 | 0.379 | 0 | 3 | 3.47 | 1.59 | 1 | 11 | 25.0% | 17.7% | 18.6% | 19.8% | 18.9% | 38.1% | 61.9% | 3318 |
| Zimbabwe | 0.64 | 0.85 | 0 | 6 | 0.32 | 0.592 | 0 | 4 | 2.59 | 1.32 | 1 | 18 | 20.9% | 21.2% | 18.3% | 21.8% | 17.7% | 28.4% | 71.6% | 9880 |

**Table S8a.** Predictors of number of days child was supervised by another child (Incidence Rate Ratio)

| **Country** | **Female** | | | **Age (in years)** | | | **Mother's level of education (lowest versus highest)** | | | **N** |
| --- | --- | --- | --- | --- | --- | --- | --- | --- | --- | --- |
|  | IRR | LCI | UCI | IRR | LCI | UCI | IRR | LCI | UCI |  |
| Afghanistan | 0.93 | 0.86 | 1.01 | 1.23 | 1.19 | 1.28 | 0.86 | 0.68 | 1.08 | 14127 |
| Algeria | 1.03 | 0.85 | 1.25 | 1.36 | 1.20 | 1.54 | 0.70 | 0.50 | 0.96 | 14562 |
| Bangladesh | 1.08 | 0.89 | 1.31 | 1.31 | 1.23 | 1.40 | 0.65 | 0.56 | 0.76 | 20692 |
| Barbados | 0.00 | 0.00 | 0.00 | 0.99 | 0.96 | 1.02 |  |  |  | 461 |
| Belarus | 1.08 | 0.62 | 1.87 | 1.29 | 1.04 | 1.60 | 1.03 | 0.47 | 2.28 | 3441 |
| Belize | 0.61 | 0.27 | 1.40 | 1.34 | 0.98 | 1.84 | 0.54 | 0.16 | 1.82 | 1939 |
| Benin | 1.04 | 0.93 | 1.15 | 1.23 | 1.18 | 1.29 | 0.67 | 0.53 | 0.85 | 12234 |
| Bhutan | 1.36 | 1.01 | 1.81 | 1.31 | 1.16 | 1.47 | 0.94 | 0.65 | 1.37 | 6236 |
| Bosnia and Herzegovina | 0.78 | 0.34 | 1.77 | 1.49 | 1.16 | 1.93 | 0.41 | 0.20 | 0.83 | 2293 |
| Cambodia | 1.23 | 0.87 | 1.74 | 1.26 | 1.12 | 1.43 | 0.34 | 0.22 | 0.53 | 6991 |
| Cameroon | 1.04 | 0.94 | 1.16 | 1.26 | 1.22 | 1.31 | 0.62 | 0.51 | 0.75 | 7004 |
| Central African Republic | 0.97 | 0.92 | 1.03 | 1.33 | 1.30 | 1.37 | 1.00 | 0.89 | 1.13 | 10025 |
| Chad | 0.99 | 0.93 | 1.06 | 1.08 | 1.06 | 1.09 | 0.85 | 0.73 | 0.99 | 10405 |
| Congo Democratic Republic | 0.99 | 0.92 | 1.06 | 1.13 | 1.11 | 1.15 | 0.87 | 0.76 | 1.01 | 7890 |
| Costa Rica | 1.25 | 0.67 | 2.32 | 1.12 | 0.71 | 1.77 | 1.29 | 0.60 | 2.76 | 2259 |
| Cuba | 2.25 | 0.53 | 9.61 | 1.84 | 1.23 | 2.76 | 0.30 | 0.07 | 1.31 | 5626 |
| Dominican Republic | 1.08 | 0.86 | 1.36 | 1.25 | 1.12 | 1.40 | 0.43 | 0.34 | 0.55 | 19648 |
| Egypt | 1.01 | 0.76 | 1.34 | 1.11 | 1.03 | 1.20 | 0.68 | 0.47 | 0.97 | 15843 |
| El Salvador | 0.39 | 0.25 | 0.61 | 1.18 | 1.02 | 1.37 | 0.63 | 0.20 | 2.00 | 7333 |
| Ghana | 1.00 | 0.83 | 1.21 | 1.32 | 1.23 | 1.42 | 0.39 | 0.28 | 0.56 | 7531 |
| Guinea-Bissau | 0.90 | 0.79 | 1.04 | 1.18 | 1.11 | 1.25 | 0.91 | 0.64 | 1.28 | 7395 |
| Guyana | 0.92 | 0.56 | 1.52 | 1.26 | 0.92 | 1.73 | 0.55 | 0.27 | 1.09 | 3351 |
| Honduras | 0.86 | 0.65 | 1.15 | 1.13 | 1.04 | 1.22 | 0.16 | 0.10 | 0.25 | 9961 |
| Iraq | 0.86 | 0.78 | 0.95 | 1.32 | 1.25 | 1.39 | 1.02 | 0.80 | 1.29 | 36299 |
| Jamaica | 0.89 | 0.31 | 2.57 | 1.13 | 0.67 | 1.91 | 0.49 | 0.07 | 3.48 | 1632 |
| Jordan | 1.42 | 0.94 | 2.14 | 1.23 | 1.15 | 1.33 | 0.60 | 0.29 | 1.23 | 10284 |
| Kazakhstan | 0.95 | 0.59 | 1.55 | 1.50 | 1.31 | 1.72 | 0.78 | 0.55 | 1.11 | 5503 |
| Kosovo under UNSC res. 1244 | 1.45 | 0.72 | 2.91 | 1.05 | 0.83 | 1.34 | 0.50 | 0.20 | 1.28 | 1646 |
| Kyrgyzstan | 0.90 | 0.67 | 1.20 | 1.33 | 1.14 | 1.56 | 0.73 | 0.45 | 1.20 | 4559 |
| Lao People's Democratic Republic | 0.81 | 0.72 | 0.92 | 1.38 | 1.30 | 1.46 | 0.64 | 0.48 | 0.86 | 10807 |
| Macedonia | 1.79 | 0.77 | 4.17 | 0.99 | 0.57 | 1.71 | 2.15 | 0.57 | 8.13 | 1359 |
| Madagascar | 0.96 | 0.81 | 1.13 | 1.49 | 1.38 | 1.62 | 1.10 | 0.75 | 1.60 | 2982 |
| Malawi | 0.99 | 0.93 | 1.06 | 1.46 | 1.43 | 1.49 | 0.73 | 0.66 | 0.81 | 18905 |
| Mali | 0.96 | 0.87 | 1.05 | 1.31 | 1.25 | 1.36 | 0.77 | 0.62 | 0.96 | 16061 |
| Mauritania | 0.93 | 0.83 | 1.03 | 1.21 | 1.15 | 1.26 | 0.64 | 0.56 | 0.73 | 8852 |
| Mexico | 0.94 | 0.60 | 1.46 | 1.23 | 1.05 | 1.44 | 0.55 | 0.35 | 0.88 | 8054 |
| Moldova | 0.98 | 0.58 | 1.66 | 1.41 | 1.21 | 1.66 | 0.93 | 0.51 | 1.69 | 1864 |
| Mongolia | 1.08 | 0.87 | 1.33 | 1.13 | 1.05 | 1.23 | 0.85 | 0.63 | 1.14 | 6045 |
| Montenegro | 1.16 | 0.39 | 3.45 | 1.38 | 1.05 | 1.80 | 1.04 | 0.08 | 13.32 | 1417 |
| Myanmar | 0.93 | 0.72 | 1.20 | 1.26 | 1.18 | 1.34 | 0.41 | 0.28 | 0.59 | 4664 |
| Nepal | 1.06 | 0.87 | 1.30 | 1.31 | 1.20 | 1.43 | 0.40 | 0.30 | 0.52 | 5307 |
| Nigeria | 1.03 | 0.97 | 1.10 | 1.40 | 1.37 | 1.43 | 0.97 | 0.88 | 1.06 | 24855 |
| Palestine | 1.01 | 0.88 | 1.15 | 1.28 | 1.21 | 1.36 | 0.68 | 0.55 | 0.84 | 7813 |
| Panama | 0.84 | 0.43 | 1.66 | 1.11 | 0.84 | 1.47 | 0.10 | 0.04 | 0.24 | 5794 |
| Rwanda | 0.98 | 0.93 | 1.03 | 1.15 | 1.12 | 1.18 | 0.62 | 0.48 | 0.80 | 7580 |
| Sao Tome and Principe | 0.84 | 0.64 | 1.12 | 1.10 | 0.98 | 1.24 | 0.60 | 0.36 | 1.00 | 1991 |
| Serbia | 0.99 | 0.38 | 2.59 | 1.77 | 0.90 | 3.48 | 0.43 | 0.07 | 2.66 | 2718 |
| Sierra Leone | 0.91 | 0.80 | 1.04 | 1.50 | 1.43 | 1.58 | 0.82 | 0.66 | 1.03 | 8290 |
| Somalia | 0.97 | 0.86 | 1.09 | 1.30 | 1.22 | 1.39 | 0.63 | 0.45 | 0.89 | 4607 |
| St Lucia | 0.68 | 0.21 | 2.18 | 0.60 | 0.28 | 1.27 |  |  |  | 292 |
| Suriname | 1.06 | 0.67 | 1.69 | 1.29 | 1.09 | 1.53 | 1.15 | 0.41 | 3.21 | 3224 |
| Swaziland | 0.96 | 0.79 | 1.17 | 1.13 | 0.94 | 1.37 | 1.04 | 0.77 | 1.41 | 2679 |
| Thailand | 0.79 | 0.54 | 1.16 | 1.51 | 1.31 | 1.76 | 0.68 | 0.42 | 1.11 | 9687 |
| Togo | 1.25 | 1.11 | 1.41 | 1.11 | 1.06 | 1.17 | 0.81 | 0.69 | 0.95 | 6611 |
| Tunisia | 1.01 | 0.74 | 1.40 | 1.34 | 1.25 | 1.45 | 0.56 | 0.41 | 0.75 | 2889 |
| Turkmenistan | 6.66 | 0.71 | 62.82 | 0.90 | 0.58 | 1.40 | 0.13 | 0.02 | 0.97 | 3757 |
| Ukraine | 1.00 | 0.67 | 1.50 | 1.42 | 1.19 | 1.68 | 0.47 | 0.35 | 0.63 | 4376 |
| Uruguay | 1.66 | 0.56 | 4.95 | 1.75 | 0.83 | 3.69 | 0.54 | 0.26 | 1.09 | 1600 |
| Vietnam | 1.22 | 0.80 | 1.88 | 1.35 | 1.16 | 1.56 | 0.39 | 0.23 | 0.65 | 3318 |
| Zimbabwe | 0.95 | 0.86 | 1.06 | 1.32 | 1.24 | 1.41 | 0.78 | 0.67 | 0.91 | 9880 |

**Table S8b.** Predictors of number of days child was supervised by another child (Incidence Rate Ratio)

| **Country** | **Number of children aged 10 - 14 years living in the household** | | | **Number of girls aged 10 - 14 years living in the household** | | | **Number of adults aged 15 years and older living in the household** | | | **Wealth score  (lowest versus highest)** | | | **Rural** | | | **N** |
| --- | --- | --- | --- | --- | --- | --- | --- | --- | --- | --- | --- | --- | --- | --- | --- | --- |
|  | IRR | LCI | UCI | IRR | LCI | UCI | IRR | LCI | UCI | IRR | LCI | UCI | IRR | CI | UCI |  |
| Afghanistan | 1.07 | 1.03 | 1.12 | 1.03 | 0.98 | 1.09 | 1.03 | 1.01 | 1.05 | 0.74 | 0.58 | 0.94 | 1.97 | 1.68 | 2.32 | 14127 |
| Algeria | 1.39 | 1.19 | 1.61 | 1.48 | 1.18 | 1.87 | 0.99 | 0.92 | 1.06 | 0.91 | 0.57 | 1.43 | 1.22 | 0.81 | 1.85 | 14562 |
| Bangladesh | 1.16 | 1.02 | 1.31 | 1.19 | 1.04 | 1.35 | 0.85 | 0.80 | 0.92 | 0.68 | 0.48 | 0.96 | 0.64 | 0.52 | 0.78 | 20692 |
| Barbados |  |  |  |  |  |  |  |  |  |  |  |  | 0.00 | 0.00 | 0.00 | 461 |
| Belarus | 1.40 | 1.20 | 1.64 | 1.89 | 0.90 | 3.98 | 0.99 | 0.74 | 1.31 | 1.46 | 0.47 | 4.55 | 2.17 | 1.21 | 3.87 | 3441 |
| Belize | 0.94 | 0.60 | 1.48 | 0.65 | 0.25 | 1.69 | 0.87 | 0.63 | 1.21 | 0.54 | 0.12 | 2.43 | 11.21 | 2.92 | 43.10 | 1939 |
| Benin | 1.09 | 1.04 | 1.14 | 1.16 | 1.07 | 1.25 | 1.01 | 0.97 | 1.05 | 0.45 | 0.37 | 0.55 | 1.37 | 1.20 | 1.55 | 12234 |
| Bhutan | 1.17 | 1.04 | 1.32 | 1.36 | 1.16 | 1.60 | 0.90 | 0.81 | 1.00 | 0.45 | 0.29 | 0.69 | 1.96 | 1.28 | 3.01 | 6236 |
| Bosnia and Herzegovina | 2.87 | 1.72 | 4.79 | 2.11 | 1.06 | 4.19 | 0.79 | 0.58 | 1.08 | 0.45 | 0.06 | 3.08 | 0.94 | 0.52 | 1.72 | 2293 |
| Cambodia | 1.50 | 1.26 | 1.78 | 1.50 | 1.19 | 1.90 | 0.58 | 0.50 | 0.67 | 0.45 | 0.25 | 0.83 | 3.38 | 2.21 | 5.16 | 6991 |
| Cameroon | 1.05 | 0.98 | 1.13 | 1.08 | 0.97 | 1.19 | 1.01 | 0.98 | 1.04 | 0.22 | 0.16 | 0.30 | 1.90 | 1.63 | 2.20 | 7004 |
| Central African Republic | 1.06 | 1.02 | 1.10 | 1.15 | 1.08 | 1.22 | 1.00 | 0.97 | 1.03 | 0.83 | 0.69 | 1.00 | 1.14 | 1.04 | 1.24 | 10025 |
| Chad | 1.06 | 1.01 | 1.11 | 1.07 | 1.02 | 1.13 | 1.02 | 1.00 | 1.05 | 0.90 | 0.74 | 1.11 | 1.02 | 0.91 | 1.16 | 10405 |
| Congo Democratic Republic | 1.11 | 1.07 | 1.15 | 1.06 | 0.99 | 1.13 | 0.94 | 0.90 | 0.99 | 0.49 | 0.37 | 0.65 | 1.98 | 1.73 | 2.26 | 7890 |
| Costa Rica | 0.74 | 0.40 | 1.36 | 1.25 | 0.55 | 2.84 | 1.05 | 0.72 | 1.53 | 0.34 | 0.05 | 2.14 | 0.72 | 0.29 | 1.79 | 2259 |
| Cuba | 1.36 | 0.43 | 4.29 | 1.54 | 0.54 | 4.40 | 1.13 | 0.85 | 1.50 |  |  |  | 0.74 | 0.25 | 2.20 | 5626 |
| Dominican Republic | 2.05 | 1.74 | 2.41 | 2.02 | 1.57 | 2.58 | 0.72 | 0.62 | 0.84 | 0.44 | 0.26 | 0.73 | 0.90 | 0.68 | 1.20 | 19648 |
| Egypt | 1.22 | 1.01 | 1.47 | 1.31 | 1.01 | 1.71 | 0.94 | 0.81 | 1.10 | 0.72 | 0.32 | 1.66 | 1.76 | 1.22 | 2.55 | 15843 |
| El Salvador | 1.77 | 1.49 | 2.11 | 2.12 | 1.48 | 3.05 | 0.88 | 0.68 | 1.12 | 0.46 | 0.27 | 0.80 | 2.27 | 1.23 | 4.18 | 7333 |
| Ghana | 1.00 | 0.91 | 1.10 | 1.06 | 0.93 | 1.22 | 1.10 | 1.05 | 1.16 | 0.23 | 0.10 | 0.54 | 1.89 | 1.28 | 2.79 | 7531 |
| Guinea-Bissau | 1.01 | 0.91 | 1.12 | 0.94 | 0.81 | 1.08 | 0.89 | 0.86 | 0.93 | 0.70 | 0.51 | 0.97 | 0.83 | 0.69 | 0.99 | 7395 |
| Guyana | 1.50 | 1.12 | 2.01 | 1.63 | 1.15 | 2.33 | 0.88 | 0.72 | 1.07 | 0.12 | 0.02 | 0.60 | 3.11 | 1.09 | 8.82 | 3351 |
| Honduras | 1.26 | 1.07 | 1.47 | 1.20 | 0.93 | 1.53 | 0.83 | 0.69 | 0.99 | 0.14 | 0.03 | 0.55 | 1.85 | 1.15 | 3.00 | 9961 |
| Iraq | 1.22 | 1.12 | 1.34 | 1.26 | 1.14 | 1.39 | 1.00 | 0.96 | 1.04 | 1.02 | 0.76 | 1.37 | 2.68 | 2.16 | 3.33 | 36299 |
| Jamaica | 0.80 | 0.37 | 1.72 | 1.62 | 0.69 | 3.83 | 0.65 | 0.50 | 0.85 | 1.13 | 0.04 | 30.73 | 0.31 | 0.05 | 2.19 | 1632 |
| Jordan | 1.26 | 1.12 | 1.43 | 1.41 | 1.17 | 1.70 | 1.11 | 0.92 | 1.33 | 0.36 | 0.14 | 0.93 | 0.58 | 0.33 | 1.02 | 10284 |
| Kazakhstan | 1.65 | 1.26 | 2.16 | 1.58 | 1.14 | 2.19 | 0.74 | 0.59 | 0.93 | 1.53 | 0.64 | 3.67 | 3.16 | 2.11 | 4.73 | 5503 |
| Kosovo under UNSC res. 1244 | 0.81 | 0.59 | 1.13 | 0.91 | 0.58 | 1.45 | 0.72 | 0.60 | 0.86 | 1.10 | 0.38 | 3.22 | 0.86 | 0.42 | 1.73 | 1646 |
| Kyrgyzstan | 1.40 | 1.11 | 1.78 | 1.09 | 0.75 | 1.57 | 0.67 | 0.50 | 0.90 | 0.93 | 0.41 | 2.10 | 0.96 | 0.53 | 1.73 | 4559 |
| Lao People's Democratic Republic | 1.12 | 1.03 | 1.22 | 1.14 | 0.99 | 1.32 | 0.90 | 0.84 | 0.98 | 0.67 | 0.38 | 1.15 | 2.50 | 1.95 | 3.20 | 10807 |
| Macedonia | 0.80 | 0.37 | 1.74 | 1.12 | 0.37 | 3.36 | 0.98 | 0.63 | 1.53 | 0.01 | 0.00 | 0.14 | 4.64 | 0.93 | 23.24 | 1359 |
| Madagascar | 1.22 | 1.09 | 1.36 | 1.25 | 1.03 | 1.53 | 1.05 | 0.95 | 1.16 | 1.20 | 0.72 | 2.01 | 1.87 | 1.19 | 2.93 | 2982 |
| Malawi | 1.18 | 1.14 | 1.21 | 1.15 | 1.10 | 1.20 | 0.99 | 0.96 | 1.02 | 0.70 | 0.58 | 0.85 | 1.85 | 1.49 | 2.29 | 18905 |
| Mali | 1.03 | 0.99 | 1.08 | 1.05 | 0.98 | 1.13 | 1.01 | 0.99 | 1.02 | 0.75 | 0.50 | 1.12 | 1.36 | 1.04 | 1.77 | 16061 |
| Mauritania | 1.10 | 1.00 | 1.21 | 1.07 | 0.92 | 1.26 | 0.98 | 0.94 | 1.02 | 0.69 | 0.49 | 0.97 | 1.06 | 0.90 | 1.25 | 8852 |
| Mexico | 1.80 | 1.52 | 2.14 | 2.27 | 1.66 | 3.09 | 1.03 | 0.89 | 1.20 | 0.71 | 0.27 | 1.87 | 1.10 | 0.76 | 1.58 | 8054 |
| Moldova | 2.18 | 1.43 | 3.32 | 2.62 | 1.50 | 4.58 | 0.86 | 0.68 | 1.08 | 0.34 | 0.14 | 0.84 | 0.86 | 0.52 | 1.42 | 1864 |
| Mongolia | 1.59 | 1.35 | 1.87 | 1.66 | 1.34 | 2.05 | 0.74 | 0.64 | 0.85 | 0.93 | 0.53 | 1.63 | 1.74 | 1.39 | 2.20 | 6045 |
| Montenegro | 1.20 | 0.75 | 1.93 | 1.48 | 0.77 | 2.82 | 0.79 | 0.45 | 1.39 | 0.71 | 0.01 | 36.71 | 0.19 | 0.06 | 0.55 | 1417 |
| Myanmar | 1.45 | 1.25 | 1.67 | 1.82 | 1.46 | 2.27 | 0.84 | 0.76 | 0.93 | 0.32 | 0.12 | 0.82 | 2.47 | 1.39 | 4.36 | 4664 |
| Nepal | 1.31 | 1.15 | 1.50 | 1.33 | 1.13 | 1.57 | 0.86 | 0.78 | 0.96 | 0.18 | 0.08 | 0.40 | 2.23 | 1.47 | 3.39 | 5307 |
| Nigeria | 1.04 | 1.00 | 1.08 | 1.03 | 0.96 | 1.10 | 1.03 | 1.00 | 1.06 | 1.01 | 0.83 | 1.23 | 1.34 | 1.16 | 1.56 | 24855 |
| Palestine | 1.44 | 1.34 | 1.55 | 1.63 | 1.47 | 1.81 | 0.98 | 0.92 | 1.05 | 0.85 | 0.59 | 1.22 | 1.06 | 0.86 | 1.31 | 7813 |
| Panama | 1.70 | 1.44 | 2.01 | 2.03 | 1.45 | 2.85 | 1.03 | 0.87 | 1.20 | 1.51 | 0.44 | 5.16 | 4.76 | 2.48 | 9.14 | 5794 |
| Rwanda | 1.26 | 1.17 | 1.36 | 1.29 | 1.17 | 1.42 | 0.88 | 0.84 | 0.92 | 0.66 | 0.49 | 0.89 | 1.68 | 1.43 | 1.97 | 7580 |
| Sao Tome and Principe | 1.29 | 1.10 | 1.53 | 1.37 | 1.04 | 1.79 | 0.95 | 0.83 | 1.10 | 0.32 | 0.19 | 0.55 | 1.11 | 0.74 | 1.68 | 1991 |
| Serbia | 2.92 | 1.25 | 6.80 | 1.07 | 0.32 | 3.61 | 0.76 | 0.51 | 1.13 | 0.07 | 0.01 | 0.40 | 5.62 | 1.87 | 16.86 | 2718 |
| Sierra Leone | 1.06 | 0.99 | 1.13 | 1.08 | 0.98 | 1.19 | 1.04 | 1.00 | 1.08 | 1.04 | 0.76 | 1.43 | 1.11 | 0.95 | 1.31 | 8290 |
| Somalia | 1.08 | 1.01 | 1.16 | 1.12 | 1.02 | 1.23 | 1.01 | 0.96 | 1.05 | 0.53 | 0.40 | 0.70 | 1.17 | 1.00 | 1.37 | 4607 |
| St Lucia |  |  |  |  |  |  | 1.15 | 0.77 | 1.69 |  |  |  |  |  |  | 292 |
| Suriname | 0.89 | 0.73 | 1.08 | 0.87 | 0.57 | 1.33 | 0.71 | 0.54 | 0.93 | 0.59 | 0.12 | 2.84 | 1.08 | 0.51 | 2.27 | 3224 |
| Swaziland | 1.06 | 0.89 | 1.26 | 0.99 | 0.72 | 1.34 | 1.10 | 1.00 | 1.20 | 0.62 | 0.28 | 1.36 | 2.32 | 1.31 | 4.10 | 2679 |
| Thailand | 1.58 | 1.17 | 2.13 | 1.29 | 0.82 | 2.04 | 0.86 | 0.76 | 0.98 | 0.20 | 0.09 | 0.46 | 1.08 | 0.73 | 1.60 | 9687 |
| Togo | 1.11 | 1.03 | 1.19 | 1.09 | 0.99 | 1.21 | 1.01 | 0.96 | 1.06 | 0.61 | 0.37 | 1.00 | 1.20 | 1.03 | 1.41 | 6611 |
| Tunisia | 1.79 | 1.49 | 2.16 | 1.95 | 1.52 | 2.50 | 0.88 | 0.74 | 1.05 | 0.74 | 0.42 | 1.31 | 1.27 | 0.83 | 1.95 | 2889 |
| Turkmenistan | 1.40 | 0.54 | 3.65 | 1.20 | 0.29 | 5.00 | 0.99 | 0.73 | 1.35 | 0.31 | 0.15 | 0.64 | 0.69 | 0.18 | 2.65 | 3757 |
| Ukraine | 2.55 | 2.06 | 3.16 | 3.30 | 2.56 | 4.24 | 1.06 | 0.87 | 1.29 | 0.31 | 0.15 | 0.64 | 1.88 | 1.27 | 2.80 | 4376 |
| Uruguay | 1.95 | 1.22 | 3.12 | 2.60 | 1.11 | 6.12 | 0.46 | 0.34 | 0.62 | 3.17 | 1.06 | 9.43 | 1.86 | 0.58 | 5.99 | 1600 |
| Vietnam | 1.09 | 0.70 | 1.68 | 1.57 | 1.04 | 2.37 | 0.69 | 0.56 | 0.85 | 0.17 | 0.07 | 0.41 | 2.23 | 1.41 | 3.51 | 3318 |
| Zimbabwe | 1.09 | 1.01 | 1.18 | 1.06 | 0.95 | 1.20 | 0.90 | 0.85 | 0.95 | 0.35 | 0.21 | 0.59 | 3.22 | 2.68 | 3.86 | 9880 |
